# Microglia/macrophages require vitamin D signaling to restrain neuroinflammation and brain injury in a murine ischemic stroke model

**DOI:** 10.1186/s12974-023-02705-0

**Published:** 2023-03-08

**Authors:** Pan Cui, Wanting Lu, Junjie Wang, Fei Wang, Xiyue Zhang, Xiaodan Hou, Fang Xu, Yan Liang, Guoliang Chai, Junwei Hao

**Affiliations:** 1 Department of Neurology, Xuanwu Hospital, National Center for Neurological Disorders, Capital Medical University, Beijing, 100053 China; 2 Department of Neurology, The First Affiliated Hospital of Zhengzhou University, Zhengzhou, 450007 Henan China; 3 Beijing Municipal Geriatric Medical Research Center, Beijing, China; 4 Department of Neurology, Tianjin Neurological Institute, Tianjin Medical University General Hospital, Tianjin, 300052 China; 5 Key Laboratory for Neurodegenerative Diseases of Ministry of Education, Beijing, China

**Keywords:** Acute ischemic stroke, Vitamin D deficiency, Vitamin D receptor, Microglia/macrophages, Neuroinflammation

## Abstract

Vitamin D deficiency is associated with worse clinical outcomes after ischemic stroke; nevertheless, the pathophysiological mechanisms remain largely unexplored. In this study, we characterized the molecular mechanisms of how vitamin D signaling modulated stroke progression in male mouse ischemia–reperfusion stroke models. We found that vitamin D receptor (VDR) exhibited a predominant upregulation in peri-infarct microglia/macrophages following cerebral ischemia. Conditional *Vdr* inactivation in microglia/macrophages markedly augmented infarct volumes and neurological deficits. VDR-deficient microglia/macrophages exhibited a more primed proinflammatory phenotype with substantial secretion of TNF-α and IFN-γ. These inflammatory cytokines further enhanced CXCL10 release from endothelial cells and blood–brain barrier disruption, and ultimately infiltration of peripheral T lymphocytes. Notably, blocking TNF-α and IFN-γ significantly ameliorated stroke phenotypes in *Vdr* conditional knockout mice. Collectively, VDR signaling in microglia/macrophages plays a crucial role in restraining ischemia-elicited neuroinflammation and stroke progression. Our findings delineate a novel mechanism underlying the association between vitamin D deficiency and poor stroke outcomes, and underline the significance of maintaining a functional vitamin D signaling in the management of acute ischemic stroke.

**Supplementary Information:**

The online version contains supplementary material available at 10.1186/s12974-023-02705-0.

## Introduction

Stroke represents the second most common cause of death and morbidity worldwide. Acute ischemic stroke is known to account for approximately 84% of prevalent strokes [1]. Following the initial ischemic insult, excitotoxicity, oxidative stress, and neuroinflammation are immediately triggered within the central nervous system (CNS), with the latter being central to secondary brain injury. As the dominant immunocompetent cells residing in the CNS, microglia constantly monitor the local environment to maintain brain homeostasis under steady-state conditions, and instantly respond to various damage-associated signals as the first line of defense. Upon activation following cerebral ischemia, microglia/macrophages exert both beneficial and detrimental effects on CNS. They engulf and eliminate dead cell debris, but also trigger a cascade of inflammatory responses by secreting large amounts of inflammatory mediators (e.g., cytokines, chemokines, and reactive oxygen species), impairing blood–brain barrier (BBB) integrity, and recruiting peripheral immune cells, thereby culminating in secondary damage to the ischemic and surrounding brain tissues [2].

It is becoming increasingly appreciated that vitamin D deficiency, an unrecognized global epidemic, is associated with increased risks and unfavorable outcomes of a variety of diseases, including cardiovascular diseases, cancer, autoimmune diseases, and neurological disorders [3]. Notably, multiple large observational studies and meta-analysis revealed a significant association between vitamin D deficiency and adverse cerebrovascular events, including an increased risk of ischemic stroke [4–8] and poor stroke prognosis [9–11]. Several preclinical studies revealed that 8-week vitamin D-deficient diet exacerbated ischemic brain injury and worsened stroke recovery in stroke rodents through excessive neuroinflammation, oxidative stress, and increased BBB leakage [12, 13]. Furthermore, mounting data have elucidated the treatment efficacy of adequate vitamin D supplementation in relieving ischemic brain injury in stroke models [14–17]. Clinically, several small trials suggested that high-dose vitamin D treatment could relieve disease severity in acute ischemic stroke patients with vitamin D inadequacy [18–20]. Despite its crucial roles in ischemic stroke, how vitamin D exerts its neuroprotective functions remains unknown.

In addition to governing calcium and phosphorus metabolism, vitamin D exerts pleiotropic non-calcemic effects, involving immunomodulation, neuroprotection, inhibition of oxidative stress, and modulation of proliferation and apoptosis [21]. 1,25(OH)_2_D, the bioactive form of vitamin D, exerts its physiological functions mainly through binding with nuclear vitamin D receptor (VDR). Upon vitamin D binding, VDR translocates into the nucleus, where it functions as a transcription factor by forming a heterodimer with the retinoid X receptor which binds to vitamin D response elements (VDRE) in the promoter regions of target genes [21]. Genetic variants in *VDR* were also found to be associated with susceptibility to ischemic stroke [22].

VDR is widely expressed in a variety of tissues [3], and is constitutively expressed in neurons and glia in the brain and exhibits elevated expression under pathological conditions [13, 14, 23–25]. In this study, we discovered that VDR in microglia/macrophages was pronouncedly upregulated in response to ischemia. VDR-deficient microglia/macrophages exhibit higher expression of cytokines TNF-α and IFN-γ, encouraging endothelial cells (ECs) to secrete more chemokine CXCL10, resulting in disrupted BBB, increased infiltration of peripheral T lymphocytes, exacerbated brain injury, and greater functional impairments. These findings provide key mechanistic explanations for how vitamin D/VDR signaling protects the brain by reducing secondary damage following acute ischemic stroke.

## Materials and methods

### Animals

Adult *Vdr*^*f/f*^ and *Cx3cr1*^*CreER*^ (#020940, Jackson Laboratory) mice of C57BL/6 background were mated to generate *Vdr*^*f/f*^*Cx3cr1*^*CreER/*+^ mice. *Vdr*^*f/f*^ mice were kindly provided by Professor James C. Fleet from Purdue University. Mice were housed under specific pathogen-free conditions with sufficient food and water. Vitamin D and progesterone have been shown to play synergistic roles in relieving acute brain injury following cerebral ischemia [26, 27]. Hence, to control the confounding impact of endogenous estrogen and progesterone, male adult animals were primarily included as the studied subjects for in vivo experiments. Male *Vdr*^*f/f*^*Cx3cr1*^*CreER/*+^ mice were used as *Vdr* conditional knockout (*Vdr*-cKO) group, and age matched male *Vdr*^*f/f*^ mice were used as controls for in vivo experiments. The mice (8–10 weeks old) were given tamoxifen (75 mg/kg/d, Sigma) dissolved in corn oil intraperitoneally (i.p.) for five consecutive days 2 weeks before the middle cerebral artery occlusion (MCAO) procedure. Neonatal mice were used to obtain primary cells for in vitro experiments. Animal numbers for all experiments are indicated in the figure legends. All experimental protocols completely adhered to ARRIVE (Animal Research: Reporting of In Vivo Experiments) guidelines and were approved by the Animal Care and Use Committee of Xuanwu Hospital (AEEI-2021-295). Adhering to the ARRIVE guidelines, we here designated neurobehavioral functions and infarct volumes as the primary endpoints.

### MCAO procedure

Focal brain ischemia was induced by transient intraluminal MCAO using a monofilament. Animals were first anesthetized with 2.5% avertin (20 µl/g, i.p.). A midline ventral neck skin incision was made, the temporal muscle was retracted, and the left carotid artery was exposed and isolated. After the common carotid artery and the external carotid artery were ligated, a silicone-coated monofilament (Doccol, Sharon, MA, USA) was inserted into the left internal carotid artery through an incision in the common carotid artery until it reached the origin of the left MCA where mild resistance was encountered (9–10 mm). One hour after blocking the MCA, the monofilament was withdrawn to restore the blood flow, and the skin was sutured. Transcranial laser-Doppler flowmetry (PeriMed) was utilized to monitor the regional cerebral blood flow (CBF) and ensure successful ischemia and reperfusion. MCA occlusion was determined by a reduction of more than 70% in CBF compared to that at baseline. The body temperature of each mouse was maintained at 37 °C ± 0.5 °C with an electric blanket pad throughout the procedure. No significant differences were noted between *Vdr*-cKO and control mice in regional CBF at baseline, during ischemia and reperfusion (Additional file 1: Fig. S2G, H), as well as in body temperature and blood pressure during MCAO or sham procedure. Mice were returned to the cages after regaining consciousness.

### Functional evaluation

Mice were assessed for neurological functions on day 1, 3, and 7 after the MCAO procedure, by corner turning test, the modified Neurological Severity Score (mNSS), foot fault test, and Rotarod test. All the outcome evaluation was conducted by independent investigators blinded to the group allocation to minimize bias. The mNSS scoring system quantified performance on motor, sensory, reflex, and balance functions, with higher scores indicating more severe neurological impairment. The corner turning test was conducted to assess behavior performance regarding sensorimotor and postural asymmetries. Mice were placed into a 30° corner joined by two connected board walls, where they could rear and turn either left or right to leave the corner. Ten trials were repeated for each mouse to calculate the percentage of left turns. The foot fault test was performed to assess sensorimotor function by calculating the percentage of foot faults out of total footsteps. Briefly, mice were placed in a grid and allowed to move freely for 5 min. A foot fault was recorded when the limb slipped through the grid hole or rested with the grid at wrist level. The Rotarod test was performed to evaluate balance and motor coordination. Mice were conditioned on a rotating rod that accelerated from 4 to 40 rpm within 300 s, which were repeated three times with 5-min intervals. Latency to fall off the rotating rod was automatically recorded up to 300 s. Data were calculated as mean values from three trials.

To minimize the experiment bias, all the neurobehavioral tests were performed on the same day, in the same mouse coding order of the same batch of animals, and also in the same experiment order, that is, mNSS score, corner turning test, foot fault test, and Rotarod test. Of note, each test was separated by at least 30-min intervals, which allowed for a sufficient rest for experimental animals.

### Infarct volume calculation

On day 3 after MCAO, mice were euthanized with an overdose of 2.5% avertin (30 µl/g, i.p.). After perfusion with chilled phosphate buffered saline (PBS, pH 7.4), the brain was rapidly removed and manually sliced into 2-mm-thick coronal sections. Rostro-caudal sections from each brain were incubated into 2% 2,3,5-triphenyltetrazolium chloride (TTC, Sigma) solution for 20 min at 37 °C in the dark. The white-appearing area represented the infarcted tissue, and the infarct volume was calculated with Image-Pro-Plus 6.0 software (Media Cybernetics, Inc., Rockville, MD USA).

### Tissue preparation

Three days after MCAO, mice were anesthetized and transcardially perfused with cold PBS. For histologic analysis, each whole brain was delicately harvested from the skull and incubated in 4% paraformaldehyde overnight at 4 °C. Following sequential dehydration in 15% and 30% sucrose, brains were embedded in Tissue-Tek® O.C.T. compound (Sakura® Finetek Inc., USA), frozen in liquid nitrogen, and stored at − 80 °C for immunofluorescence staining. After the cerebellum and olfactory bulbs were removed, the ischemic/ipsilateral hemispheres were collected, frozen and stored in liquid nitrogen until protein was extracted for western blot analysis. For quantitative real-time PCR analysis (qRT-PCR), ischemic/ipsilateral hemispheres snap-frozen in liquid nitrogen were sufficiently lysed with TRIzol Reagent (Invitrogen, USA) and stored at − 80 °C thereafter.

### Immunofluorescence staining

Frozen sections (8 µm) mounted onto poly-L-lysine-coated glass slides were subjected to immunofluorescence staining. In brief, frozen brain sections were fixed, permeabilized with 0.3% Triton for 15 min at room temperature, and washed with PBST (PBS + 0.1% Tween 20). To block non-specific staining, brain sections were incubated in 3% bovine serum albumin (BSA) in PBST for 1 h at room temperature. Subsequently, the sections were incubated in diluted primary antibodies in a humidified chamber overnight at 4 °C, then incubated with species-specific Alexa Fluor (488, 594, 546 or 647)-conjugated secondary antibodies (Thermo Scientific, USA) for 1 h at room temperature in the dark. The following primary antibodies were used in this experiment: mouse anti-VDR (1:200, Santa Cruz), rabbit anti-Iba1 (1:500, Wako), rabbit anti-glial fibrillary acidic protein (GFAP) (1:1000, Abcam), rabbit anti-NeuN (1:1000, Abcam), rat anti-CD31 (1:50, BD Biosciences), rat anti-CD31 (1:50, BD Biosciences), rat anti-CD16/32 (1:500, BD Biosciences), rabbit anti-CD3 (1:100, Abcam), mouse anti-CXCL10 (1:50, Abcam), rabbit anti-PDGFRβ (1:100, Abcam), rabbit anti-Claudin-5 (1:100, Abcam), and rabbit anti-NF-κB (1:400, Cell Signaling Technology). After nuclear counterstaining with DAPI (Abcam, ab104139), the sections were visualized using a confocal microscope (Zeiss, Germany) or a fluorescence microscope (Nikon, Tokyo, Japan). To evaluate ischemia-induced apoptosis within the brain, brain sections were stained with TUNEL reagents using a TUNEL kit (Beyotime, China). The infarct core was identified as an area in which the majority of DAPI-stained nuclei were shrunken. The peri-infarct area was determined as the region 200–300 µm distant from the infarct border where Iba1^+^ microglia/macrophages are abundantly accumulated. Immunofluorescence intensity was quantified using Image Pro Plus6.0 (Media Cybernetics, Inc. Rockville, MD). Image quantitative analyses were conducted on one or two random microscopic fields in the interested area of each section. Two sections were assessed per sample.

### qRT-PCR

Total RNA was extracted from fresh brain tissues using TRIzol reagent as the protocol instructed. Total RNA of each sample was determined using a NanoDrop 2000 spectrophotometer (Thermo Scientific, Germany). Total RNA (2 µg) was reverse-transcribed in a 20 µl reaction volume using TransScript First-Strand cDNA Synthesis Super Mix (TransGen Biotech, China). Then, cDNA was amplified by FastStart Universal SYBR Green Master (Roche, Germany) on a Bio-Rad CFX96 Detection System (Bio-Rad, USA). Sequences of primers used in qPCR are displayed in Additional file 1: Table S1. All qPCR reactions were repeated in triplicate, and all quantitative data were presented as the relative expression of the interested genes normalized to that of *Gapdh* using the 2^−ΔΔCt^ comparative method.

### Western blot analysis

Tissue samples were immersed in protein lysis buffer containing protease and phosphatase inhibitor cocktail (Roche, Germany), and homogenized by sonication or with a homogenizer (DHS, Q24RC, China). Concentrations of extracted total proteins were quantified using a bicinchoninic acid protein (BCA) assay kit (Thermo Scientific, USA). An equal amount of protein from each sample was electrophoretically separated by SDS-PAGE, and then transferred to PVDF membranes (Merck). After blocked with 5% non-fat milk in TBST washing buffer (TBS + 0.1% Tween 20) for 1 h at room temperature, the membranes were incubated with the following diluted primary antibodies overnight at 4 °C: mouse anti-VDR (1:500, Santa Cruz), rabbit anti-NF-κB p65 (1:1000, Cell Signaling Technology), rabbit anti-phospho-NF-κB p65 (1:1000, Cell Signaling Technology), rabbit anti-TNF-α (1:1000, Abcam), mouse anti-GAPDH (1:2000, ZSGB-Bio), and mouse anti-β-actin (1:2000, ZSGB-Bio). Then, they were incubated with species-specific secondary antibodies (1:5000) conjugated to horseradish peroxidase for 1 h at room temperature. The membranes were rinsed with ECL substrate (Millipore), and the protein bands were visualized on a Bio-Rad Gel Doc Imager (Bio-Rad, USA). The intensity of each band relative to that of the housekeeping genes was measured using densitometry (ImageJ software). The relative expression levels of proteins were normalized against GAPDH or β-actin.

### Preparation of single-cell suspensions

To quantify the counts of immune cell subpopulations in the brain and spleen using fluorescence-activated cell sorting analysis (FACS), single-cell suspensions of brain or spleen tissues were prepared. After mice were anesthetized, the spleens and brains were quickly removed before and after transcardial perfusion with cold PBS, respectively, and then were immersed in cold PBS. Spleens and brains were mechanically homogenized through 70-µm cell strainers (BD Biosciences), and centrifuged. To isolate brain cells, 5 ml of 30% Percoll (GE Healthcare, Sweden) was added to the cell pellets obtained from brain tissues, which were centrifuged at 700×*g* for 10 min. After the myelin layer and the supernatant were carefully aspirated, the remaining pellets were washed with PBS, and resuspended into single-cell suspensions for FACS. A total of 7 ml Red Blood Cell Lysis Buffer (Solarbio, China) was added to spleen samples for erythrocyte lysis. Finally, single-cell suspensions of splenocytes were prepared.

### FACS analysis

Fresh brain- and spleen-derived single-cell suspensions at a density of 10^5^–10^6^ cells/100 µl in 1% BSA/PBS were incubated with mouse antigen-specific antibodies conjugated with one type of fluorochrome, including FITC, PE, APC, Percp-Cy5.5, APC/cy7, PE/Cy7, AF700, and BV421. Generally, immune cell subsets in the brain and spleen were identified by extracellular staining with specific antibodies against the following antigens: CD11b, CD45, Ly6G, F4/80, CD86, CD206, CD3, CD4, CD8a, CD19, and NK1.1. For intracellular staining of IFN-γ, TNF-α, and CXCL10, cells were stimulated for 4 h with 1X cell activation cocktail with Brefeldin A (BioLegend). After incubation, cells were stained for specific surface molecules. Following fixation and permeabilization using a fixation/permeabilization kit (BD Biosciences), staining for intracellular cytokines was performed. Intracellular staining for CXCL10 was performed using an unconjugated CXCL10 antibody (Abcam) and an Alexa Fluor 488-conjugated secondary antibody (Invitrogen). Antibody staining was conducted in accordance with the manufacturer’s instructions. Finally, samples were acquired on FACS Aria III flow cytometer (BD Bioscience) and analyzed using FlowJo software.

### Annexin V-propidium iodide (PI) apoptosis assay

FACS was also used to measure cell apoptosis in brain tissues using the Annexin V-PI apoptosis assay (Solarbio, China) according to the manufacturer’s instructions. Briefly, isolated brain cells were washed with binding buffer, and then resuspended into a density of 10^5^ cells/100 µl in binding buffer. Next, cell suspensions were stained with 5 µl Annexin V-FITC for 10 min at room temperature in the dark, and then with 5 µl PI for 5 min under the same conditions. Finally, 400 µl PBS was added for FACS within 1 h. Annexin V^+^ PI^+^ cells were identified as apoptotic cells.

### Multiplex chemokine immunoassay

We measured the chemokine concentrations in brain homogenates using LEGENDplex™ 13-plex proinflammatory chemokine panel (740451, BioLegend) according to the manufacturer’s instructions. Briefly, brain tissues were freshly dissected from animals after perfusion with cold PBS and were immediately frozen in liquid nitrogen. Then, the frozen brain tissues were homogenized in cold-PBS. The tissue supernatants were collected after centrifugation for 10 min at 12,000 rpm. The samples were next diluted in assay diluent buffer, and concentrations of chemokines were measured. The 13-plex mouse proinflammatory chemokine panel allows simultaneous quantification of 13 chemokines including CXCL9, CXCL10, CCL3, CCL4, CXCL13, CXCL5, CCL22, CCL5, CCL20, CCL11, CCL17, CXCL1, and CCL2. After incubation with the APC-conjugated capture beads and PE-conjugated detection reagents, the samples were analyzed on FACS Aria III and quantified using the LEGENDplex™ software, version 8.0. Chemokine concentrations of each sample were adjusted by the total protein concentrations as measured by BCA Protein Assay Kit.

### Isolation of microglia/macrophage from adult brain tissue

Brain single-cell suspensions were prepared from poststroke/sham *Vdr-*cKO and control mice 3 days after the procedure. For in vitro microglial IFN-γ induction, brain single-cell suspensions were stained with APC-conjugated anti-CD45 and BV421-conjugated anti-CD11b antibodies. CD11b^+^CD45^int^ microglia were sorted using FACS Aria III. For RNA-sequencing analysis of microglia/macrophages, brain single-cell suspensions were purified by magnetic cell separation (MACS) using CD11b magnetic microbeads (Miltenyi Biotec). In brief, single-cell suspensions of 10^7^ cells were resuspended into a 90 μl MACS buffer. In accordance with the manual’s recommended procedures, CD11b microbeads (10 μl/10^7^ cells) were incubated to label microglia. Afterwards, a MACS column was used to attach CD11b microbead-labeled microglia/macrophages. Finally, the purity of sorted microglia/macrophages was confirmed by flow cytometric analysis after sorting (> 98%).

### RNA-sequencing

Total RNA was extracted from microglia/macrophages isolated from the ischemic brains of *Vdr*-cKO and *Vdr*^*f/f*^ mice using TRIzol reagent. Microglia/macrophages were purified by MACS using CD11b microbeads (as described above). High-throughput RNA-sequencing was performed by CapitalBio Corporation (Beijing, China). Qubit fluorometer (Invitrogen) was used to evaluate the quantity and quality of extracted RNA samples. Agilent 2100 BioAnalyzer (Agilent Technologies, USA) and RNase-free agarose gel electrophoresis were employed to determine RNA integrity. Total RNA from each sample of sufficient quality, defined as RNA integrity number > 7.0, was included for subsequent analysis. In brief, poly-A-containing mRNA purified from the total RNA from each sample was employed for cDNA library construction. Next, cDNA libraries were sequenced on an Illumina NovaSeq6000 sequencer (Illumina), the quality of which was evaluated with Fast QC (Version 0.11.5). The parameters for defining significantly differentially expressed genes (DEGs) were a ≥onefold change in transcript abundance and a false discovery rate-corrected *P* < 0.05. For analysis of the DEGs associated with disease-associated microglia signature and IFN-stimulated gene sets, the gene list was taken from the literature [28, 29]. Hierarchical clustering heatmaps of the DEGs were drawn using an R package to display the differential expression pattern across samples. Furthermore, the DEGs were subjected to non-redundant GO functional annotation and Reactome pathway enrichment analysis using the online Webgestalt (http://www.webgestalt.org/option.php). Raw RNA-sequencing data for samples in this study were uploaded to the Gene Expression Omnibus database (http://www.ncbi.nlm.nih.gov/geo/) under the accession number GSE190171.

### Oxygen glucose deprivation (OGD)

Ischemia was simulated in vitro by OGD that was induced by replacing the growth medium with pre-warmed DMEM without serum and glucose (Gibco, Invitrogen, 11966-025). Cells were then transferred into a humidified chamber (Stem Cell, 27310) flushed with a gas mixture of 95% N2 and 5% CO2, and was maintained in a 37 °C incubator. To mimic the in vivo ischemia/reperfusion process, complete culture medium was recovered, and cells were returned into the normoxic incubator at the appropriate time depending on cell types. For instance, 1-h OGD was performed in primary microglia and neurons, while 4-h OGD was applied in cell lines, including BV2 microglial cells and brain-derived Endothelial cells.3 (bEnd.3 cells), as previously suggested [30, 31].

### Cell isolation, culture and treatment

Primary microglia and neurons were prepared from 1-day-old neonatal mice. Briefly, neonatal mouse brain cortices were dissected and placed in ice-cold HBSS without Ca^2+^ and Mg^2+^. After the meninges on the surface of the cortices were sufficiently removed, the cortices were ground and digested using 0.125% trypsin and DNase I for 20 min at 37 °C. Following termination of the enzymatic digestion using complete media, cell suspensions were filtered through a 70 µm cell strainer. For the culture of primary microglia, cells were centrifuged and resuspended in DMEM/F12 supplemented with 10% fetal bovine serum (FBS) (Gibco), 1% penicillin/streptomycin (Gibco), and 5 ng/ml M-CSF (Sigma) and plated into flasks precoated with poly-D-lysine (Solarbio, China). After mixed glial cultures reached complete confluence on day 10, microglia were detached from the mixed glial cultures by spinning the flasks at 200 rpm for 4 h at 37 °C in an orbital shaker. The microglia-enriched culture supernatants were collected and cultured for 24 h to allow adhesion for subsequent experiments. The purity of isolated microglia was quantified by FACS. To induce Cre-loxP recombination and excise VDR, microglia isolated from neonatal *Vdr*-cKO and *Vdr*^*f/f*^ mice were treated with 4-hydroxytamoxifen (0.02 mg/ml) for 48 h.

For the culture of primary neurons, resuspended cells were seeded into complete high-glucose DMEM media for 4 h, and then cultured in neurobasal medium (Thermo Scientific, USA) supplemented with 2% B27, 1% HEPES, 1% glutamine, and 1% penicillin/streptomycin for 1 week, during which half of the medium was replaced every 3 days. To mimic in vivo hypoxic/ischemic conditions, primary neural cells underwent 1-h OGD. Twenty-four hours after returning into the normal culture conditions, the conditioned media (CM) collected from primary neurons, together with 100 ng/ml IL-12 and 100 ng/ml IL-18, were supplemented as inducers of microglial IFN-γ production.

The murine BV2 microglial cells were cultured in Dulbecco’s modified Eagle’s medium (DMEM, Gibco) supplemented with 10% FBS and 1% penicillin/streptomycin in 5% CO2 at 37 °C. To induce VDR functional overexpression, cells were cultured in media supplemented with 10^–7^ M 1,25(OH)_2_D. Primary brain mouse ECs were purchased from Mingzhoubio (MZ-M0115, Ningbo, China). A commonly used EC line, bEnd.3 (ATCC), was cultured in complete DMEM.

For neutralizing TNF-α and/or IFN-γ signaling in vitro, 300 ng/ml anti-TNF-α antibody (50349-RN023, Sino Biological, China) and/or 5 µg/ml anti-IFN-γ antibody (505834, BioLegend) were administered.

### Short hairpin RNA (shRNA)-mediated knockdown of VDR

BV2 cells transfected with recombined lentivirus vectors containing specific shRNA targeting *Vdr* (sh*Vdr*; 5ʹ-CGTGGACATTGGCATGATGAA-3ʹ) and non-specific negative control shRNA (shNC; 5ʹ-TTCTCCGAACGTGTCACGTAA-3 ʹ), which were designed and provided by Hanbio Biotechnology (Shanghai, China). Briefly, BV2 cells were seeded into 24-well plates (5 × 10^4^ cells/well). When the cells reached 30–50% confluence the culture medium was replaced with infection medium containing 5 µg/ml polybrene and lentiviral particles at a multiplicity of infection of 20. After 24 h, the transfection medium was replaced by complete medium. Cells were examined under a fluorescence microscope after 72 h of transfection, and EGFP positive cells were considered successfully transfected. Furthermore, BV2 cells with decreased VDR expression were selected with puromycin (2 µg/ml, Sigma); the efficiency of VDR knockdown was determined via western blot analysis.

### Transwell co-culture system

To investigate the biological effects of microglia on the secretion of CXCL10 from brain ECs in vitro, we co-cultured microglia with brain ECs. Briefly, primary microglia or BV2 cells were seeded onto the upper chamber of a 12-mm transwell culture plate (0.4-µm pore size, polycarbonate membrane, Corning, NY, USA), and primary brain ECs or bEnd.3 cells were plated onto the bottom chamber. Upon cells reached confluence, the co-culture system was subjected to OGD. Afterwards, cell culture supernatants and ECs in the bottom well were collected to evaluate CXCL10 secretion using ELISA and FACS, respectively. In addition, the co-culture media in the lower chamber were used as CM for T cell migration assay.

### T cell isolation and in vitro migration assay

Splenic T cells were isolated from male 10- to 12-week-old C57/BL6 mice using anti-CD3-conjugated magnetic beads (Miltenyi Biotec). The purity of sorted CD3^+^ T cells was > 90% as analyzed by FACS. Isolated T cells were first stimulated with soluble anti-CD3 (5 µg/ml) and anti-CD28 (5 µg/ml) for 72 h in RPMI-1640 medium supplemented with 10% FBS, 1% penicillin/streptomycin, 2-mercaptoethanol and L-glutamine. A total of 1 × 10^6^ T cells in 100 µl RPMI containing 1% FBS were seeded onto 6.5-mm transwell inserts (3 µm pore size, polyester membrane, Corning, Corning, NY, USA), and the inserts were placed into the CM collected from the microglia–ECs co-culture system. Transwell plates were maintained in a 37 °C incubator for 16 h, and T cell migration was quantified by counting the number of cells that migrated to the lower compartment.

### ELISA

According to the manufacturer’s guidelines (Invitrogen), the levels of TNF-α, IFN-γ, and CXCL10 in the culture supernatants were evaluated using a Mouse TNF-α ELISA Kit, a Mouse IFN-γ ELISA Kit, and a Mouse CXCL10 ELISA Kit, respectively.

### In vivo antibody treatment

For neutralizing TNF-α signaling in vivo, we performed intraperitoneal injections of 500 µg anti-TNF-α-clone XT3.11 (#BE0058, BioX Cell) at 3 h after MCAO. For blockade of IFN-γ, mice were treated intraventricularly (i.c.v.) with 1 µg Ultra-LEAF™ Purified anti-mouse IFN-γ antibody (505834, BioLegend) at 12 h after MCAO. Similar amounts of rat IgG (#BE0088, clone HRPN, BioXcell or 400431, BioLegend) were delivered via corresponding route as isotype control antibodies.

### Statistical analysis

For behavioral assays, a power analysis on the basis of preliminary data was conducted on each group to determine appropriateness of sample size (G power). No priori statistical methods were used to predetermine sample size for other experiments. Sample sizes for each specific experiments were indicated in the figure legends. Experiments and analysis were performed in a blinded manner. Specifically, for experiments prone to subjectivity during data collection, including poststroke infarct volume calculation and neurological evaluation, the experimenters were blinded to grouping. Meanwhile, all data quantification and comparison were also performed in a blinded manner, that is, analyzers were blinded to grouping.

Statistical analysis was performed using GraphPad Prism 8.0 software. Data are expressed as mean ± SD. Differences were considered statistically significant when *P* < 0.05. Unpaired Student's *t*-test was used for two-group comparison for continuous variables with normal distribution. One-way ANOVA followed by appropriate post hoc tests was used for multiple-group comparison. For multi-factor comparison, two-way ANOVA followed by appropriate post hoc tests was performed. If data were not normally distributed, Mann–Whitney test was performed for two-group comparison, and Kruskal–Wallis test followed by Dunn’s post hoc test was performed for multiple-group comparison.

## Results

### Cerebral ischemia induces VDR upregulation in microglia/macrophages

**Fig. 1:**
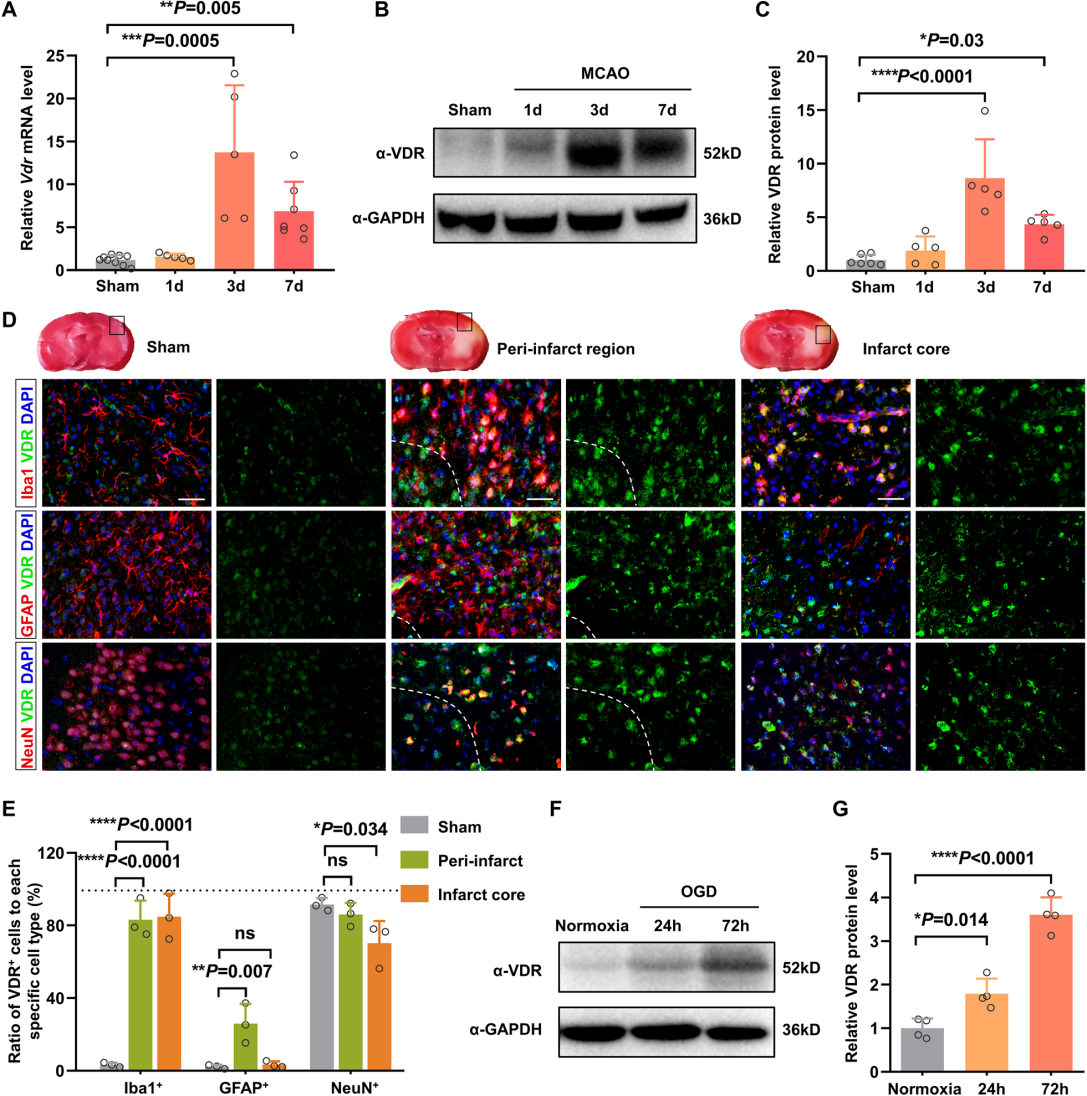
VDR expression is robustly upregulated in microglia/macrophages after cerebral ischemia. **A** qRT-PCR analysis of the relative *Vdr* mRNA expression in the sham brain and ischemic brain of mice on day 1, 3, and 7 after the surgery (*n* = 9:5:5:7). **B**, **C** Quantification of VDR protein in the sham and ischemic brain by taking sham brain as 1.0 (*n* = 6:5:5:5). **D**, **E** Representative immunofluorescence images of VDR with Iba1, GFAP, and NeuN in the sham brain cortex, peri-infarcted regions, and infarct cores of mice 3 days after MCAO (**D**). Dashed lines divide the infarction core and ischemic penumbra. Scale bar, 40 µm. **E** Shows the percentage of VDR^+^ cells among Iba1^+^, GFAP^+^, and NeuN^+^ cells, respectively (*n* = 3 per group). **F**, **G** Quantification of VDR protein in normoxia controls and OGD-treated primary microglia taking normoxia as 1.0 (*n* = 4 per group). Normoxia represented a “sham” control to OGD. Each symbol represents one biological replicate. Data are expressed as mean ± SD. Kruskal–Wallis test with Dunn’s post hoc test for **A**. One-way ANOVA with Dunnett’s post hoc test for **C**, **E**, and **G**. *P < 0.05, **P < 0.01, ***P < 0.001, ****P < 0.0001

To investigate the role of vitamin D signaling in acute cerebral ischemia, we examined VDR expression in the brains of mice subjected to transient middle cerebral artery occlusion (MCAO). Compared to the sham group, both mRNA and protein levels of VDR were significantly increased in the ischemic hemispheres of the MCAO group, with a peak on day 3 and a subsequent decline on day 7 after ischemia (Fig. 1A–C). In the sham brain cortex, VDR immunoreactivity was predominantly observed in neurons and was barely detectable in astrocytes and resting ramified microglia (Fig. 1D, E). In contrast, VDR immunoreactivity was abundantly enhanced in peri-infarct microglia/macrophages and surrounding astrocytes 3 days after MCAO. Consistently, the proportion of VDR^+^ microglia/macrophages at the margins of infarction increased from 3.2% in sham-operated controls, to 22.1% and 80.5% in poststroke mice on day 1 and day 3, respectively (Additional file 1: Fig. S1). The percentage of VDR^+^ astrocytes increased significantly but to a lesser extent (2.2% to 25.9%), and no significant increase was observed in neurons. The proportion of VDR^+^ microglia/macrophages within the infarct core also showed a comparable increase to that within the peri-infarct regions 3 days after cerebral ischemia (Fig. 1D, E).

Additionally, we isolated primary microglia from neonatal wild-type mice and subjected them to 1-h oxygen–glucose deprivation (OGD) followed by re-oxygenation, which mimicked brain ischemia. Western blot analysis also confirmed the substantial upregulation of VDR expression in primary microglia after 72 h (Fig. 1F, G). Together, these results demonstrated that cerebral ischemia elicited prominent VDR upregulation in microglia/macrophages, suggesting a potential role of vitamin D signaling in the pathogenesis of acute ischemic stroke.

### Conditional genetic deletion of VDR in microglia/macrophages leads to a worse outcome after stroke

**Fig. 2:**
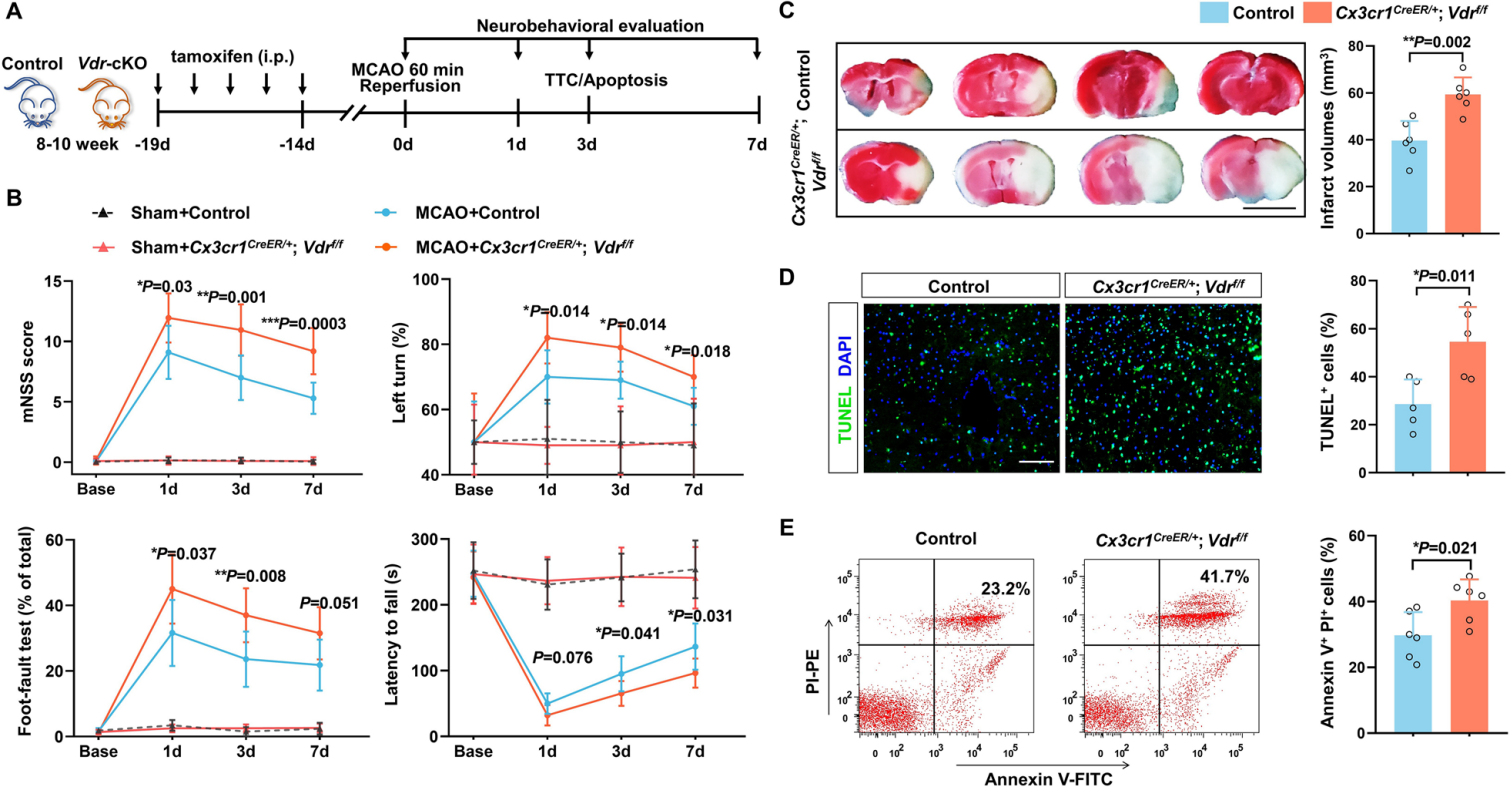
*Vdr* conditional knockout in microglia/macrophages worsens poststroke functional outcome. **A** An overview of the experimental design. **B** Sensorimotor deficits were evaluated using the mNSS score (top left), corner turning test (top right), foot fault test (bottom left), and Rotarod test (bottom right) in *Vdr*^*f/f*^ control and *Vdr-*cKO mice 1, 3 and 7 days after MCAO (*n* = 10 per group). **C** TTC staining of brain sections from control and *Vdr-*cKO mice 3 days after MCAO. Four representative rostro-caudal brain sections are displayed (*n* = 6 per group). Scale bar, 5 mm. **D** TUNEL immunostaining indicates the number of apoptotic cells in the peri-infarct area of ischemic brains of indicated groups (*n* = 5 per group). Scale bar, 100 µm. **E** FACS analysis of brain apoptotic cells of control and *Vdr-*cKO mice using an Annexin-PI kit (*n* = 6 per group). Each symbol represents one mouse. Data are expressed as mean ± SD. *P < 0.05, **P < 0.01, ***P < 0.001, *Vdr-*cKO versus control group by two-way repeated measures ANOVA with Greenhouse–Geisser correction followed by Sidak's post hoc test for **B**, and two-tailed Student’s *t* test for **C**, **D**, and **E**

To determine whether VDR signaling in microglia/macrophages would have a specific impact on stroke progression and outcome, we generated inducible *Vdr* conditional knockout (*Vdr-*cKO: *Cx3cr1*^*CreER/*+^; *Vdr*^*f/f*^) mice by crossing *Vdr*^*f/f*^ mice with *Cx3cr1*^*CreER*^ mice (Additional file 1: Fig. S2A). MCAO procedure was performed 2 weeks after 5 consecutive days of tamoxifen administration (Fig. 2A). Considering the relatively low VDR expression in microglia/macrophages under homeostatic conditions, we confirmed the efficacy of cKO of VDR expression in poststroke *Vdr-*cKO and littermate *Vdr*^*f/f*^ control mice. Immunostaining of brain slices of controls showed pronounced VDR expression in microglia/macrophages 3 days after MCAO, which was almost absent in *Vdr-*cKO mice (Additional file 1: Fig. S2B), indicating an efficient conditional VDR ablation.

Under physiological conditions, *Vdr-*cKO mice did not exhibit any significant growth deficit, as evaluated by body weight changes (Additional file 1: Fig. S2C), nor any defects in the number of splenic immune cells (Additional file 1: Fig. S2D, E), as well as cerebral microglia, astrocytes, and neurons (Additional file 1: Fig. S2F). No significant differences were noted between *Vdr*-cKO and control mice in regional CBF at baseline and during ischemia (Additional file 1: Fig. S2G). In contrast, poststroke *Vdr-*cKO mice exhibited significantly more severe sensorimotor deficits in a battery of behavioral tests on day 1, day 3, and day 7 after MCAO, including the mNSS score, corner turning test, foot fault test, and Rotarod test (Fig. 2B). Notably, *Vdr-*cKO mice showed significantly larger infarcts than controls 3 days after MCAO (Fig. 2C), accompanied by larger amounts of apoptotic cells as revealed by TUNEL and Annexin V staining (Fig. 2D, E). Additionally, we replicated the data on the infarct volumes in adult female mice. Similarly, we found an exacerbation of infarct lesions in female *Vdr-*cKO mice compared to controls (Additional file 1: Fig. S3), though females exhibited lesser infarct volumes than those of genotype-matched males, probably owing to the protective roles of estrogen. Together, these findings indicated that VDR signaling in microglia/macrophages is essential for restraining brain injury and functional deficits following cerebral ischemia.

### VDR deficiency in microglia/macrophages aggravates brain inflammatory milieu after cerebral ischemia

**Fig. 3:**
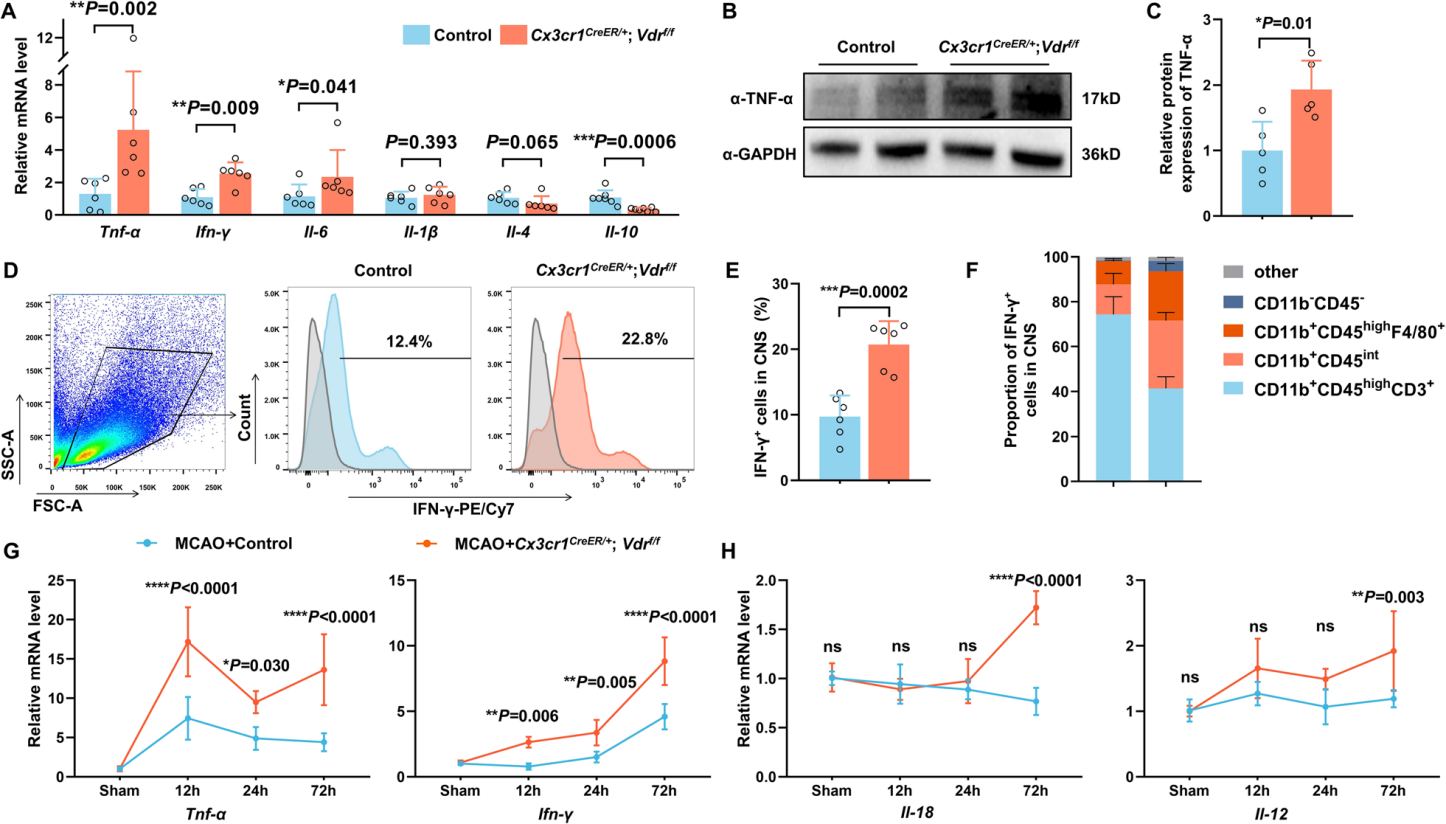
VDR ablation in microglia/macrophages intensifies CNS inflammatory milieu after cerebral ischemia. **A** qRT-PCR analysis of the relative cytokine mRNA levels within the ischemic brain of control and *Vdr-*cKO mice 3 days after MCAO (*n* = 6 per group). **B**, **C** Western blot analysis and quantification of TNF-α within the ischemic brains of indicated groups. Relative protein levels were quantified by taking controls as 1.0 (*n* = 5 per group). **D**, **E** FACS analysis of IFN-γ expression in brain tissues of control and *Vdr-*cKO mice (*n* = 6 per group). **F** The proportion of individual cellular types among IFN-γ-expressing cells in brain tissues of control and *Vdr-*cKO mice, as measured by FACS analysis (*n* = 6 per group). **G** mRNA expression changes of *Tnf-α* and *Ifn-γ* in the ischemic brain of control and *Vdr*-cKO mice within the first 3 days after MCAO (*n* = 5 per group). **H** mRNA expression changes of *Il-18* and *Il-12* in the ischemic brain of control and *Vdr*-cKO mice (*n* = 5 per group). Each symbol represents one mouse. Data are expressed as mean ± SD. *P < 0.05, **P < 0.01, ***P < 0.001, ****P < 0.0001, *Vdr-*cKO versus control group by Mann–Whitney test in **A**, two-tailed Student’s *t* test in **C**, **E**, and two-way ANOVA with Sidak's post hoc test in **G**, **H**

It has been well established that poststroke cerebral inflammatory cascades play pivotal roles in driving secondary brain injury [2]. We therefore evaluated cytokine levels in the ischemic brains of control and *Vdr-*cKO mice 3 days after MCAO by qRT-PCR. Notably, several key proinflammatory cytokines, including tumor necrosis factor α (*Tnf-α*) and interferon γ (*Ifn-γ*), were markedly increased in the ischemic hemisphere of *Vdr-*cKO mice (Fig. 3A). In contrast, *Il-10*, a crucial anti-inflammatory cytokine, showed a significant reduction. Western blot analysis also indicated an increased protein level of TNF-α in poststroke *Vdr-*cKO brains (Fig. 3B, C). Furthermore, the proportion of IFN-γ-expressing cells was markedly elevated in poststroke *Vdr-*cKO mice compared to controls (Fig. 3D, E). It has become increasingly appreciated that invading T cells were the primary source of delayed IFN-γ production in the ischemic brain, specifically at a later time point of 3 days following ischemia onset [32]. We indeed observed that more than 70% of IFN-γ-expressing cells in the ischemic brain were CD3^+^ T cells in controls. However, in poststroke *Vdr-*cKO mice, only about 40% of IFN-γ-expressing cells were CD3^+^ T cells, and surprisingly, about a third were CD11b^+^CD45^int^ resident microglia, compared to 13% in controls, and 22% were CD11b^+^CD45^high^F4/80^+^ macrophages, compared to 10% in controls (Fig. 3F). The FACS gating strategies of brain resident and infiltrating immune cells are shown in Additional file 1: Fig. S4.

Apart from upregulated TNF-α and IFN-γ 3 days after MCAO, we also noticed distinct dynamics of their expression at earlier time points (Fig. 3G). In both control and *Vdr-*cKO mice, *Tnf-α* upregulation reached its maximum as early as 12 h after MCAO, and maintained comparable levels at 24 h and 72 h. A higher level of *Tnf-α* was observed in *Vdr-*cKO mice at all these time points after MCAO, but not under sham conditions. *Ifn-γ* displayed notable upregulation as early as 12 h after MCAO in *Vdr-*cKO mice, but only at 72 h in controls. Its upregulation exhibited a time-dependent increase in both groups, in parallel with the time course of lymphocyte infiltration. Multiple evidence indicates that IL-18, synergizing with IL-12, strongly stimulates the production of IFN-γ from microglia/macrophages [33, 34]. As such, we evaluated the mRNA expression of *Il-18* and *Il-12* (Fig. 3H). We observed their delayed but evident increases on day 3 in *Vdr-*cKO mice, but not in controls. In sum, the absence of VDR in microglia/macrophages significantly heightened the ischemia-elicited inflammatory milieu in the acute phase of ischemic stroke.

### Microglia lacking VDR exhibit proinflammatory properties with excessive cytokine release after cerebral ischemia

**Fig. 4:**
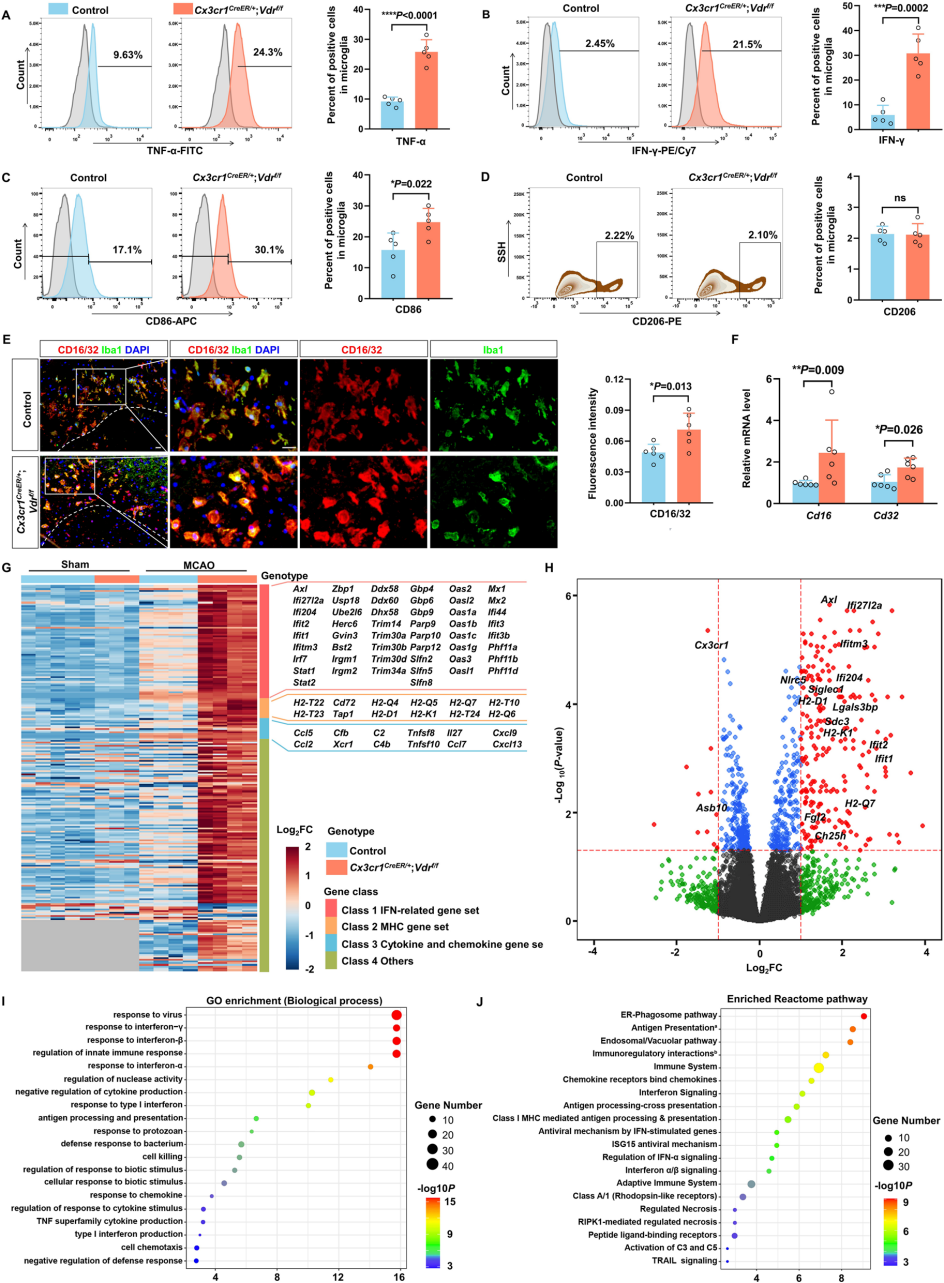
Microglia/macrophages lacking VDR exhibit more drastic proinflammatory properties after cerebral ischemia. **A**–**D** FACS analysis of the expression of TNF-α (**A**), IFN-γ (**B**), CD86 (**C**), and CD206 (**D**) in CD11b^+^CD45^int^ microglia from control and *Vdr*-cKO mice 3 days after MCAO (*n* = 5 per group). **E** Immunostaining of Iba1 and CD16/CD32 within ischemic penumbra of indicated groups. Dashed lines divide the infarction core and ischemic penumbra. Scale bar, 20 µm. The fluorescence intensity of CD16/32 was quantified (*n* = 6 per group). **F** mRNA levels of *Cd16* and *Cd32* in the ischemic brain of indicated groups (*n* = 6 per group). **G** Heatmap of differential gene expression profiles of microglia/macrophages isolated from control and *Vdr*-cKO mice undergoing sham and ischemic insult 3 days after the surgery. Differentially expressed gene is defined as adjusted *P* < 0.05 and log2-fold change (FC) > 1.0. **H** Volcano plot analysis displaying dysregulated genes related to disease-associated microglia signature performed in microglia/macrophages isolated from control and *Vdr*-cKO mice at day 3 after MCAO. **I** GO annotation results of the top 20 biological process terms based on differentially expressed genes in **G**. **J** Top 20 terms by pathway enrichment analysis using Reactome datasets. ^a^The full term is “Antigen Presentation: Folding, assembly and peptide loading of class I MHC”; ^b^the full term is “Immunoregulatory interactions between a Lymphoid and a non-Lymphoid cell”. Each symbol represents one mouse. Data are expressed as mean ± SD. *P < 0.05, **P < 0.01, ***P < 0.001, ****P < 0.0001, *Vdr-*cKO versus control group by two-tailed Student’s *t* test in **A**–**E** and Mann–Whitney test in **F**

It is known that activated microglia orchestrate both initiation and progression of inflammatory responses evoked by ischemic brain damage. We therefore assess microglial cytokine secretion and inflammatory phenotypes 3 days after cerebral ischemia by FACS analysis gating on CD11b^+^CD45^int^ microglia. In both rodent models and human patients, microglia/macrophages have been recognized as a main source of TNF-α during the early stages of cerebral ischemia [32, 35, 36]. Expectedly, *Vdr*-cKO mice had roughly 2.5 times more TNF-α-expressing microglia than controls (Fig. 4A). Following our observation that microglia are another major source of IFN-γ in *Vdr*-cKO mice, we further examined IFN-γ production in microglia and observed a substantial sixfold increase in the proportion of IFN-γ-expressing microglia in *Vdr*-cKO mice (Fig. 4B).

To confirm the impact of VDR deletion on microglial IFN-γ production, we used ELISA to quantify IFN-γ production in cultured primary microglia obtained from poststroke *Vdr-*cKO and control mice. Nevertheless, even in the presence of IL-12 and IL-18, IFN-γ concentrations in both groups were below the detection limit. We proposed that certain substances released from ischemic/hypoxic neurons, likely damage-associated molecular patterns, are required to trigger IFN-γ expression in microglia. Therefore, we stimulated IFN-γ secretion by adding the condition media (CM) from primary neuron cultures challenged by OGD. Indeed, a considerably greater amount of IFN-γ was detected from microglia lacking VDR (Additional file 1: Fig. S5A, B), indicating that VDR-deleted microglia served as an alternative source of IFN-γ following cerebral ischemia. This observation accounted for the early increase of IFN-γ in *Vdr*-cKO mice within 1 day poststroke (Fig. 3G) when infiltration of IFN-γ-producing lymphocytes is negligible.

Additionally, we assessed microglial activation on day 3 after MCAO. Despite a comparable proportion of CD206^+^ anti-inflammatory microglia, a larger proportion of primed microglia expressed proinflammatory marker CD86 was observed in the ischemic brains of *Vdr-*cKO mice (Fig. 4C, D). Both immunofluorescent staining and qRT-PCR also revealed significantly higher levels of CD16/32, another proinflammatory microglial surface marker, in poststroke *Vdr*-cKO mice (Fig. 4E, F). Overall, our findings suggest that defective VDR signaling in microglia leads to its polarization towards a proinflammatory phenotype and increased cytokine production in response to cerebral ischemia, thereby aggravating poststroke neuroinflammation and disease outcome.

### VDR deficiency renders microglia/macrophages engaged in IFN pathway activation after cerebral ischemia

To acquire mechanistic insights into how microglia/macrophages become more inflammatory in the absence of VDR following cerebral ischemia, we performed RNA-sequencing on microglia/macrophages isolated from control and *Vdr-*cKO mice undergoing sham or MCAO procedures. While behaving similarly to controls under sham settings, VDR-deleted microglia/macrophages displayed markedly different patterns of gene expression 3 days after MCAO. Notably, we observed a significant increase in the expression of IFN pathway-related genes in VDR-deficient microglia/macrophages. Among these were a large panel of IFN-stimulated genes, including *Irf7*, *Ifi27l2a*, *Usp18*, *Ube2l6*, *Ddx58*, *Ifit1*, *Mx1*, and members of *Oas* and *Parp* families. In agreement with a previous finding that IFN induces the expression of MHC class I genes involved in antigen processing and presentation [37], VDR-deficient microglia/macrophages expressed higher levels of *H2-D1*, *H2-K1*, *H2-T24* to *-T22*, *H2-Q4* to *-Q7*, and *Tap1*, as well as costimulatory molecule *Cd72* (Fig. 4G). Notably, a number of microglial hallmark genes, including *Ifitm3*, *Axl*, *Ifi27l2a*, *Ifit1*, *Ifit2, Siglec1*, *Ch25h*, *Fgl2*, *Ifi204*, and *Lgals3bp*, showed remarkable upregulation following VDR ablation, reflecting more prominent microglial disease-associated signatures (Fig. 4H)*.* STAT1, an essential transcription factor required for the activation of IFN signaling pathway, was likewise significantly increased in VDR-ablated microglia/macrophages.

We further performed pathway enrichment analysis of the differentially expressed genes in VDR-deficient microglia/macrophages. Further Gene Ontology (GO) annotation and Reactome pathway enrichment analysis revealed prominent inflammatory signaling, specifically IFN-associated signaling, antigen processing and presentation, and dysregulated secretion and responses of cytokines and chemokines (Fig. 4I, J). Together, these findings indicated critical roles of VDR in restricting phenotypic switch of microglia/macrophages towards heightened inflammatory signatures after cerebral ischemia, particularly by limiting IFN signaling activation.

### VDR deficiency in microglia/macrophages aggravates T cell infiltration via CXCL10 induction

**Fig. 5:**
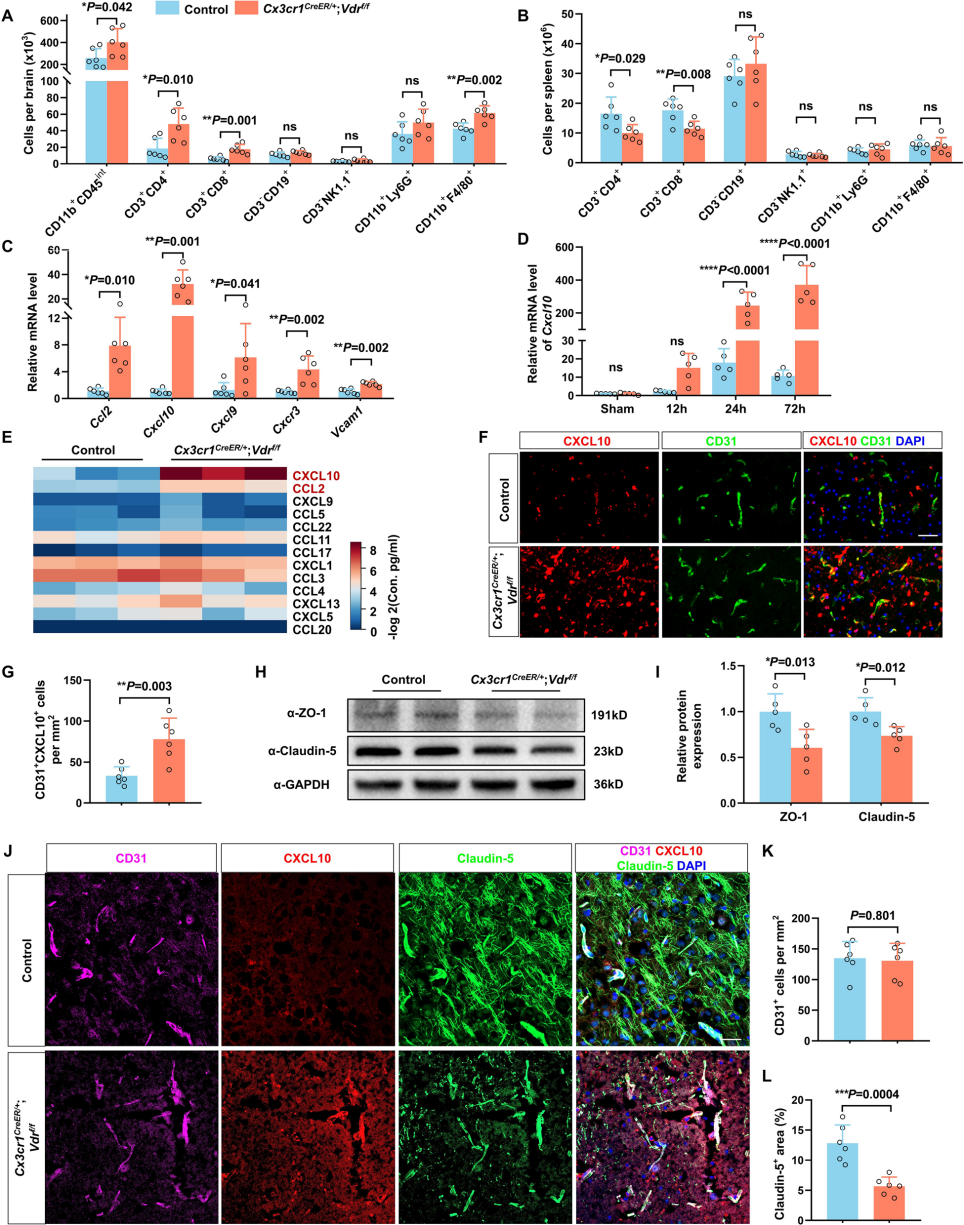
VDR deficiency in microglia/macrophages aggravates poststroke T cell CNS infiltration via CXCL10 induction. **A** The counts of resident CD11b^+^CD45^int^ microglia and brain infiltrating immune cell subsets, as evaluated by FACS analysis of brains of control and *Vdr-*cKO mice 3 days after MCAO (*n* = 6 per group). **B** FACS analysis of splenic immune cell subsets of indicated groups 3 days after MCAO (*n* = 6 per group). **C** qRT-PCR analysis of the relative mRNA levels of chemokines and adhesion molecules responsible for T cell and monocyte infiltration in the ischemic brains of control and *Vdr-*cKO mice (*n* = 6 per group) 3 days after MCAO. **D** Time-course changes of *Cxcl10* mRNA levels in the ischemic brains of control and *Vdr*-cKO mice within the first 3 days after MCAO (*n* = 5 per group). **E** Heatmap plot showing chemokine concentrations in the ischemic brains of indicated groups, as measured by multiplexed immunoassay 3 days after MCAO (*n* = 3 per group). **F**, **G** Immunofluorescence staining for CD31 and CXCL10 of brain sections of indicated groups 3 days after MCAO. Scale bar, 40 µm. The counts of CD31^+^CXCL10^+^ ECs are quantified (*n* = 6 per group). **H**, **I** Western blot analysis of ZO-1 and Claudin-5 of the ischemic brains of indicated groups 3 days after MCAO (*n* = 5 per group). **J** Immunofluorescence staining of CD31, CXCL10, and Claudin-5 of brain sections from *Vdr*-cKO and *Vdr*^*f/f*^ mice 3 days after MCAO. **K** Quantification of the counts of CD31^+^ cells of each group in **J** (*n* = 6 per group). **L** Quantification of Claudin-5^+^ areas of each group in **J** (*n* = 6 per group). Each symbol represents one mouse. Data are expressed as mean ± SD. *P < 0.05, **P < 0.01, ***P < 0.001, ****P < 0.0001, *Vdr-*cKO versus control mice by two-tailed Student’s *t* test in **A**, **B**, **G**, **I**, **K**, and **L**, Mann–Whitney test in **C**, and two-way ANOVA followed by Sidak's post hoc test in **D**

Microglial activation facilitates peripheral leukocyte infiltration into the ischemic brain, which further exacerbates brain inflammation and damage. Using FACS, we observed larger amounts of microglia in *Vdr*-cKO mice than in controls 3 days after MCAO. Furthermore, deletion of VDR in microglia/macrophages considerably favored cerebral infiltration of T lymphocytes (CD3^+^CD4^+^ and CD3^+^CD8^+^) and monocytes/macrophages (F4/80^+^) (Fig. 5A). Immunostaining of brain slices also revealed increased T cell recruitment in *Vdr-*cKO mice 3 days after MCAO (Additional file 1: Fig. S6A, B). In parallel, *Vdr-*cKO mice displayed a significant decrease in the number of splenic CD4^+^ and CD8^+^ T cells (Fig. 5B), as well as in the count of total splenocytes and the weight of spleen (Additional file 1: Fig. S6C, D).

Leukocyte recruitment substantially depends on locally elevated chemokines and endothelial cell adhesion molecules. Using qRT-PCR, we observed markedly elevated levels of inflammatory mediators in the ischemic brains of *Vdr-*cKO mice 3 days after MCAO, including *Cxcl10*, *Cxcl9*, *Cxcr3*, and *Vcam1* (Fig. 5C). CXCL10 and CXCL9 are crucial for T cell infiltration via binding to CXCR3 that is predominantly expressed on activated CD4^+^ and CD8^+^ T cells [38]. In comparison to sham conditions, higher *Cxcl10* mRNA levels were detected as early as 12 h after MCAO in *Vdr-*cKO mice and increased by over 200 folds 24 h and 72 h poststroke (Fig. 5D). Increased CXCL10 and CCL2 proteins were also confirmed in brain homogenates via multiplex immunoassay. Comparable to the magnitude in its mRNA elevation, CXCL10 concentrations, of note, were roughly 50 times more abundant in *Vdr-*cKO mice than that in controls (Fig. 5E). Immunostaining of brain slices also indicated a higher level of CXCL10 in *Vdr-*cKO mice, exhibiting a widespread extracellular distribution throughout the ischemic cores and peri-infarct regions 3 days after MCAO (Additional file 1: Fig. S7A).

Several lines of evidence indicated microglia/macrophages as a source of CXCL10 during multiple sclerosis and CNS viral infections [39, 40]. Intuitively, one might expect VDR-deleted microglia/macrophages to be the direct culprit origin of the remarkably increased CXCL10. However, CXCL10 immunostaining was barely detected in microglia/macrophages, which, instead, was predominantly observed in ischemia-affected ECs in *Vdr*-cKO mice (Additional file 1: Fig. S7B). Expectedly, we observed a larger number of CXCL10-expressing ECs compared to controls (Fig. 5F, G). Overall, these findings suggested that the sharp increase of CXCL10 was mainly from ECs rather than VDR-deleted microglia/macrophages.

Increased peripheral immune cell infiltration could also arise from BBB leakage. Indeed, we found that VDR ablation in microglia/macrophages markedly impaired BBB integrity after stroke, as evidenced by reduced expression of endothelial tight junction proteins, ZO-1 and Claudin-5, 3 days after MCAO (Fig. 5H, I). Co-labeling of CD31, CXCL10, and Claudin-5 also revealed that in the presence of a comparable density of CD31, decreased expression of Claudin-5, as well as elevated CXCL10, were found in *Vdr*-cKO mice compared to controls (Fig. 5J–L). Moreover, reduction of pericyte coverage was seen adjacent to CXCL10-expressing ECs in *Vdr-*cKO mice (Additional file 1: Fig. S7C). Taken together, VDR deletion in microglia/macrophages resulted in significantly increased chemokine secretion and decreased BBB integrity poststroke, ultimately exacerbating CNS infiltration of immune cells, particularly T lymphocytes.

### Microglia modulates endothelial CXCL10 production in a VDR-dependent manner

**Fig. 6:**
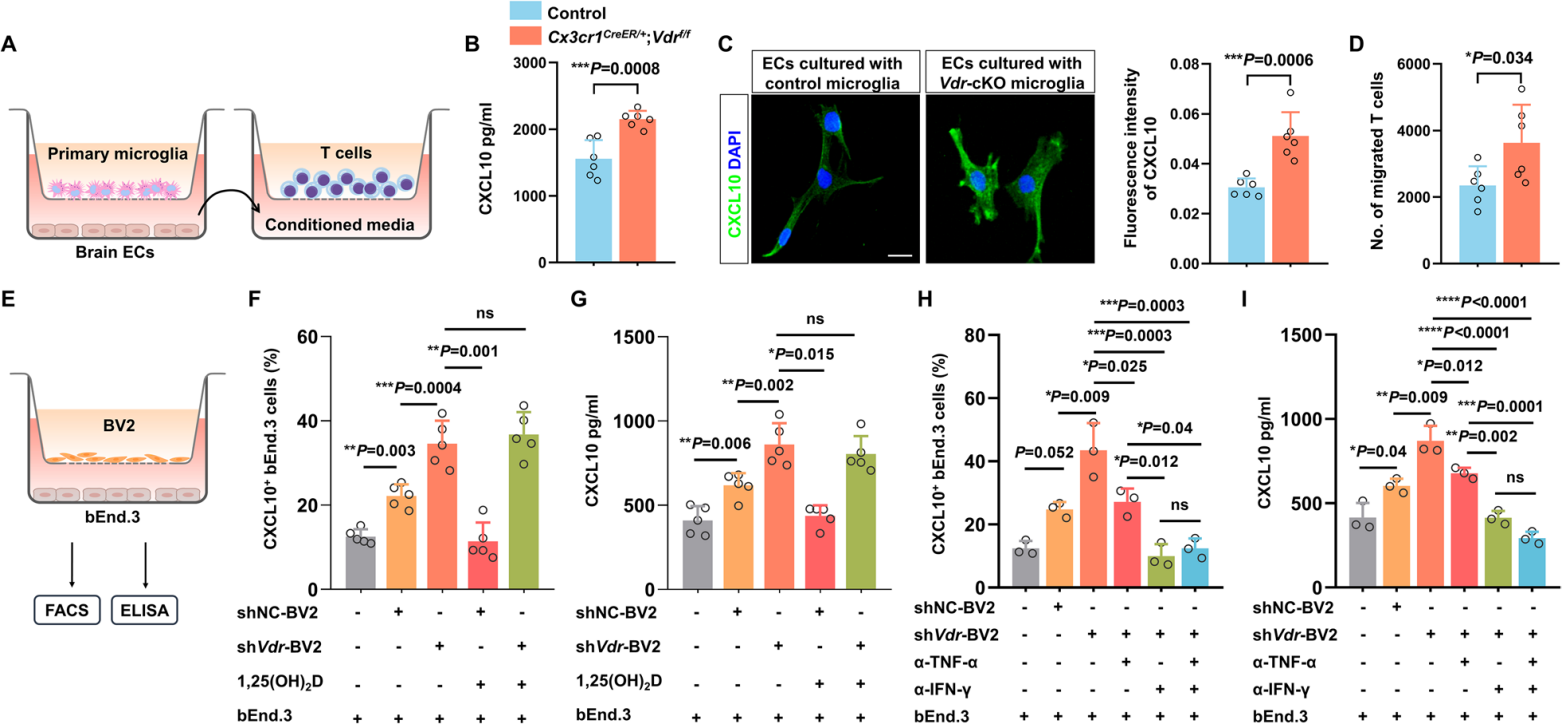
Microglia modulates endothelial CXCL10 production in a VDR-dependent manner. **A** Illustration of the in vitro co-culture and transmigration system. The former contains primary brain endothelial cells (ECs) in the lower chamber and primary microglia isolated from neonatal control and *Vdr-*cKO mice in the upper chamber. Co-culture media in the lower chamber are used as conditioned media to stimulate T cell migration in the latter transmigration system. **B** CXCL10 concentrations in the co-cultures containing control and *Vdr*-cKO microglia (*n* = 6 per group). **C** Quantification of immunofluorescence intensity of CXCL10 in co-cultured ECs of indicated groups 48 h after OGD (*n* = 6 per group). Scale bar, 20 µm. **D** The counts of T cells migrating towards the lower chamber (*n* = 6 per group). **E** Illustration of the co-culture system containing bEnd.3 and BV2 cells. **F**, **G** Quantification of CXCL10 expression in bEnd.3 cells cultured alone, co-cultured with control, VDR-deficient, or VDR-overexpressing BV2 cells, as measured by FACS (**F**) and ELISA analysis (**G**) (*n* = 5 per group). **H**, **I** Quantification of CXCL10 expression in bEnd.3 cells of indicated groups treated by anti-TNF-α and/or anti-IFN-γ antibodies, as measured by FACS (**H**) and ELISA analysis (**I**) (*n* = 3 per group). Each symbol represents one biological replicate. Data are expressed as mean ± SD. *P < 0.05, **P < 0.01, ***P < 0.001, ****P < 0.0001. Two-tailed Student’s *t* test for **B**–**D**; for the former four columns in **F**, **G**, one-way ANOVA followed by Dunnett's post hoc test was used to compare each column with the second one; for the latter two columns, two-tailed Student’s *t* test was used. For the former three columns in **H**, **I**, one-way ANOVA followed by Dunnett's post hoc test was used to compare each column with the second one; for the latter four columns, one-way ANOVA followed by Tukey's post hoc test was used to compare each column with the others

We next aimed to examine whether VDR loss in microglia was sufficient to induce CXCL10 secretion from brain ECs. Primary brain ECs were co-cultured with microglia isolated from neonatal brains of control and *Vdr*-cKO mice (Fig. 6A). Following OGD challenge, we did detect higher concentrations of CXCL10 in the co-culture media of *Vdr*-cKO group than that of controls (Fig. 6B). Similarly, the fluorescence intensity of CXCL10 was significantly higher in ECs co-cultured with VDR-deficient microglia (Fig. 6C). Furthermore, we applied the co-culture CM to another transmigration system and assessed its ability to induce T cell chemotaxis. Expectedly, a greater number of migrated T cells were observed in response to the CM from *Vdr-*cKO group (Fig. 6D).

Following that, we sought to determine whether VDR expression levels in microglia had any impact on ECs’ CXCL10 production. We then co-cultured bEnd.3 ECs with BV2 cells, in which VDR expression could be easily manipulated (Fig. 6E). As in primary microglia, VDR expression in BV2 cells was relatively low in homeostatic conditions but was significantly increased following OGD (Additional file 1: Fig. S8A, B). Transfection of *Vdr*-specific shRNA (sh*Vdr*) into BV2 cells efficiently reduced VDR expression by 82% (Additional file 1: Fig. S8C, D). On the other hand, administration of 0.1 µM 1,25(OH)_2_D, the bioactive vitamin D known to activate VDR [41], effectively stimulated a twofold increase in VDR expression (Additional file 1: Fig. S8E, F). CXCL10 concentrations, as well as the proportion of CXCL10-expressing bEnd.3 cells in BV2-bEnd.3 co-cultures, were significantly increased in the presence of VDR-deficient BV2 cells but decreased in the presence of VDR-overexpressing BV2 cells (Fig. 6F, G). Together, these results indicated that following ischemic stimuli, microglial VDR is a major determinant in regulating CXCL10 secretion from ECs.

TNF-α and IFN-γ play synergistic roles in the induction of CXCL10 [42]. As seen in in vivo VDR-deleted microglia, we also detected a significantly higher level of TNF-α in VDR-deficient BV2 cells 24 h following OGD (Additional file 1: Fig. S9A). In contrast, IFN-γ was below the ELISA detection limit, probably due to the peculiar inflammatory properties of BV2 microglial cell line. Additionally, both NF-κB p-p65 subunit expression and p65 nuclear translocation were significantly increased in VDR-deficient BV2 cells but decreased in VDR-overexpressing BV2 cells (Additional file 1: Fig. S9B–E). In the co-cultures containing VDR-deficient BV2 cells, administration of TNF-α neutralizing antibodies partially abrogated CXCL10 induction. Yet unexpectedly, IFN-γ neutralization resulted in a further reduction in CXCL10 production (Fig. 6H, I). Together, these results suggested that TNF-α and IFN-γ mediated the crosstalk between microglia and ECs during CXCL10 induction, and this inflammatory response was remarkably intensified as a result of VDR deficiency in microglia.

### Blockade of TNF-α and IFN-γ rescues exacerbated stroke phenotypes in *Vdr*-cKO mice

**Fig. 7:**
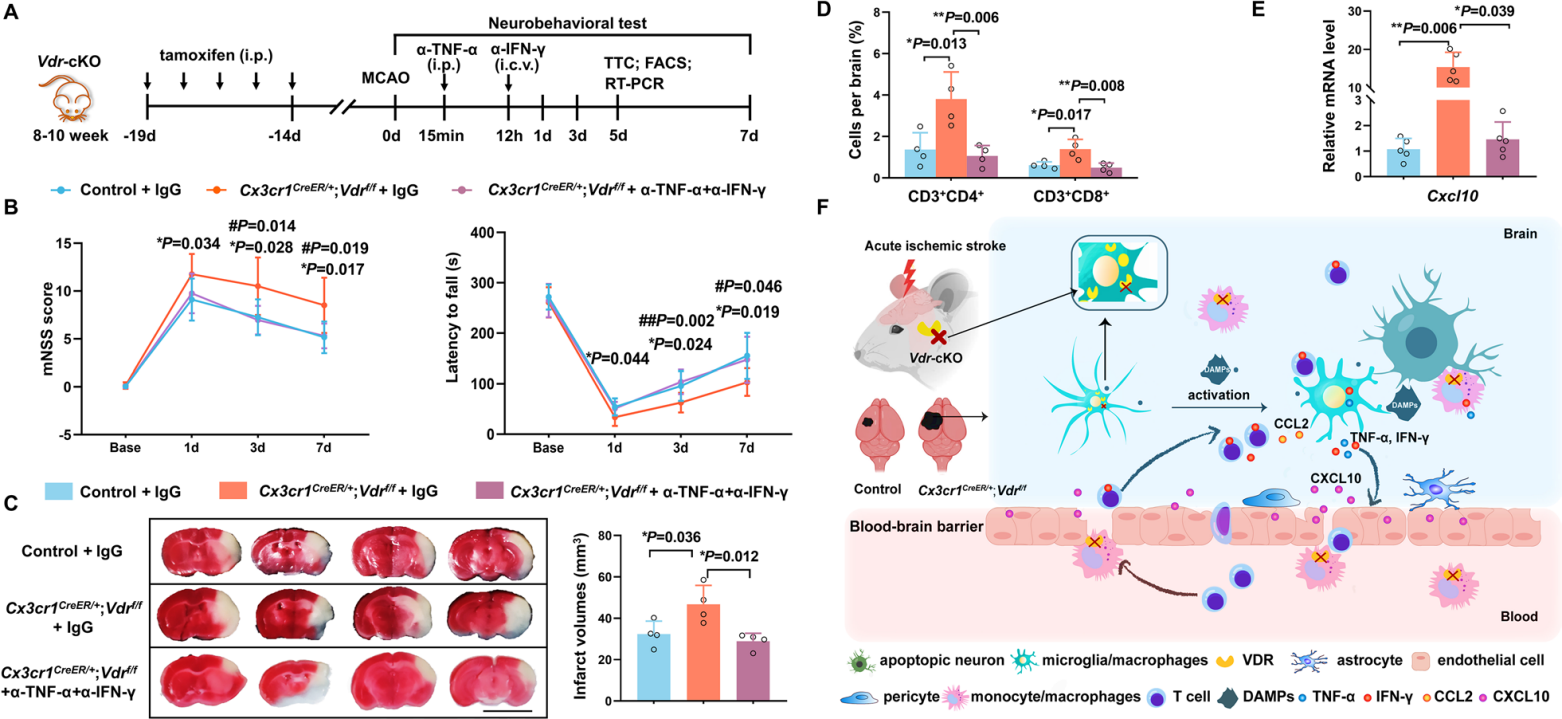
Blockade of TNF-α and IFN-γ rescues inflammatory consequences caused by VDR defects in microglia/macrophages. **A** Treatment strategies using anti-TNF-α antibodies intraperitoneally (i.p.) and anti-IFN-γ antibodies intraventricularly (i.c.v.) in stroke *Vdr-*cKO mice. **B** mNSS score (right) and Rotarod test (left) of indicated groups after MCAO (*n* = 10 per group). **C** Infarct volumes of indicated groups 5 days after MCAO (*n* = 4 per group). Scale bar, 5 mm. **D** FACS analysis of brain infiltrating CD4^+^ and CD8^+^ T cells of indicated groups 5 days after MCAO (*n* = 4 per group). **E** *Cxcl10* mRNA expression in the ischemic brains of indicated groups 5 days after MCAO (*n* = 5 per group). **F** Schematic diagram illustrating the role of microglia/macrophage VDR in acute ischemic stroke. Each symbol represents one mouse. Data are expressed as mean ± SD. * or # *P* < 0.05, ** or ##* P* < 0.01, ***P < 0.001. Two-way repeated measures ANOVA with Greenhouse–Geisser correction followed by Sidak's post hoc test for **B**, *denotes the difference between control and *Vdr*-cKO mice, # symbolizes the difference between *Vdr*-cKO mice and *Vdr*-cKO mice receiving anti-TNF-α and anti-IFN-γ antibodies; one-way ANOVA followed by Tukey's post hoc test for **C**, **D**; Kruskal–Wallis test followed by Dunn's post hoc test for **E**

Given that in vitro TNF-α and IFN-γ neutralization could reverse CXCL10 elevation and that remarkably increased TNF-α, IFN-γ and CXCL10 were detected in the ischemic brains of *Vdr*-cKO mice, we then assessed whether combined inhibition of TNF-α and IFN-γ could likewise reduce CXCL10 production and rescue the exacerbated inflammation and functional deficits in stroke *Vdr*-cKO mice. To test this, neutralizing antibodies against TNF-α and IFN-γ were administered intraperitoneally or intraventricularly, respectively, according to established protocols (Fig. 7A) [43, 44]. Compared to control IgG, antibody-based TNF-α and IFN-γ antagonization significantly improved functional outcomes and reduced infarct volumes in poststroke *Vdr*-cKO mice (Fig. 7B, C). Expectedly, decreased T cell infiltration and reduced *Cxcl10* expression were also observed (Fig. 7D, E). One would expect a more overt rescue in infarct volumes in *Vdr*-cKO mice receiving TNF-α and IFN-γ antagonization, which was supposed to be smaller than that of control mice receiving control IgG. However, only a nonsignificant trend is observed in Fig. 7C. Presumably, the massive accumulation of TNF-α and IFN-γ in the ischemic brains of *Vdr*-cKO mice might require larger doses of neutralizing antibodies to sufficiently block TNF-α and IFN-γ. Summary findings are illustrated in Fig. 7F

Together, these findings revealed that VDR-mediated signaling has a substantial impact on the proinflammatory capacity of microglia/macrophages by restricting the release of TNF-α and IFN-γ, which could further influence endothelial CXCL10 release, leukocyte infiltration, infarction evolution, and eventually functional outcomes following acute cerebral ischemia.

## Discussion

Vitamin D deficiency is a largely underestimated epidemic that frequently coexists with cardiovascular diseases [21]. Increasing population- and cohort-based studies have demonstrated an unequivocal association between vitamin D deficiency and the incidence, progression, and outcomes of acute ischemic stroke [4–11]. However, the molecular mechanisms underlying the poor prognosis of ischemic stroke patients with vitamin D deficiency remain inadequately investigated. Our study provides novel insights into the mechanism by which vitamin D-VDR signaling axis robustly impacts stroke progression by modulating microglia/macrophage-initiated neuroinflammation following cerebral ischemia. Overall, VDR is required in microglia/macrophages to restrain secondary brain damage by inhibiting ischemia-induced expression of cytokines TNF-α and IFN-γ, which could further increase production of chemokine CXCL10 by ECs and subsequent recruitment of T cells and monocytes into the CNS.

Despite recognized VDR elevation in the ischemic brain [13, 14, 23], brain cell-specific alterations in VDR expression and the resulting pathophysiological actions remain largely underappreciated. Our study showed that VDR upregulation was more pronounced in microglia/macrophages than in other CNS cell types after cerebral ischemia, suggesting a potential endogenous protective mechanism against excessive neuroinflammation induced by acute ischemic stroke. In line with this hypothesis, mice with conditional VDR deletion in microglia/macrophages exhibited exacerbated phenotypes with larger infarct sizes and worse functional outcomes following MCAO. The mechanistic underpinnings predominantly involve the drastic inflammatory cascades elicited by VDR-deficient microglia/macrophages, as evidenced by its substantially increased production of inflammatory cytokines, including TNF-α and IFN-γ.

Microglia are known to be the primary source of TNF-α in the early stages of acute ischemic stroke in both rodents and humans [32, 35, 36]. However, few studies have identified microglia/macrophages as an alternate source of IFN-γ after ischemic brain injury, though microglia were shown to produce IFN-γ during in vivo CNS parasite infection and in vitro cytokine stimulation [37, 45]. Instead, infiltrating T cells have long been recognized as the major source of IFN-γ at later stages, typically three days after cerebral ischemia [32, 44]. In the early post-ischemia phase, IFN-γ was observed to be derived primarily from a non-T-cell source, with a minor contribution from T cells [46]. Critically, the present study shows that VDR ablation confers microglia/macrophages with the ability to produce IFN-γ and make them another crucial source of IFN-γ. We hypothesized that, in the absence of VDR, microglia/macrophages significantly contribute to the initial wave of IFN-γ elevation in the ischemic brain prior to lymphocyte invasion, thereby exacerbating early ischemic brain damage. Since IFN-γ was only detected in VDR-deficient primary microglia after treatment with CM from OGD-challenged neurons, it appears that signals released from damaged neurons are necessary to stimulate IFN-γ expression in VDR-deficient microglia. It is postulated that vitamin D/VDR signaling suppresses IFN-γ-producing capacity by hindering its transcription at the level of promoter and/or silencer. Future study is required to further determine the exact molecular mechanisms underlying VDR-regulated IFN-γ expression in microglia/macrophages.

Transcriptome analysis revealed significantly upregulated gene sets in microglia/macrophages from poststroke *Vdr*-cKO mice, primarily involved in IFN pathway activation and damage-associated phenotypes. This further corroborates VDR as a key determinant in regulating the immune signatures of microglia/macrophages in stroke pathophysiology. Similar alterations in microglia/macrophages also contribute to neuroinflammation and disease progression in Alzheimer’s disease [28, 29]. Whether this gene expression pattern regulated by VDR could impact on poststroke long-term neurological sequelae, such as cognitive decline, awaits to be elaborated in future studies.

Infiltration of immune cells in response to microglial activation significantly aggravates neuroinflammation and disease progression after cerebral ischemia [47]. Remarkably, defective VDR signaling in microglia/macrophages resulted in pronounced upregulation of chemokines after acute ischemic stroke, including CXCL10 and CCL2, together with aggravated disruption of the BBB and infiltration of peripheral T cells and monocytes/macrophages. In contrast to previous studies that identified microglia/macrophages as the primary sources of CXCL10 in multiple sclerosis patients [39], we detect evident CXCL10 expression primarily in ECs, but not in VDR-deficient microglia/macrophages in the ischemic brain. Brain endothelium has been established as a crucial source of CXCL10 that allows for subsequent transendothelial leukocyte migration and recruitment in CNS disorders [48]. Possibly, CXCL10 from brain endothelial cells could act in an autocrine or paracrine manner, drive cell injuries in itself or the surrounding BBB components, and cause loss of endothelial tight junction proteins. Together, massive accumulation of chemokines and disruption of the BBB profoundly aggravated leukocyte recruitment.

Presumably, local excessive inflammatory milieu elicited by microglia/macrophage profoundly affected endothelial function in a paracrine manner. In vitro co-culture assay with microglia and ECs confirmed that VDR deficiency in microglia caused robust CXCL10 induction in ECs following OGD, whereas VDR overexpression in microglia significantly attenuated this pathological inflammatory response. CXCL10 expression is prominently regulated by TNF-α and IFN-γ [42]; therefore, it is reasonable to hypothesize that increased TNF-α and IFN-γ expression in VDR-deficient microglia/macrophages triggers increased CXCL10 production in ECs. Correspondingly, combined blockade of TNF-α and IFN-γ significantly ameliorated CXCL10 induction both in vitro and in vivo and rescued the exaggerated ischemic brain injury observed in *Vdr*-cKO mice.

Vitamin D-VDR signaling axis in microglia/macrophages is a crucial modulator of poststroke neuroinflammation and outcomes, whereas this finding did not preclude the importance of VDR in other brain cell types, such as astrocytes and neurons, during stroke pathogenesis. In addition, this study focused on the actions of microglia/macrophage VDR in the acute phase of cerebral ischemia. Their roles in chronic pathophysiological alterations remain to be elucidated in light of the association between vitamin D deficiency and stroke recurrence and poststroke cognitive deficits [49–51]. A major limitation of this study is a lack of data on ageing and female mice. Ageing significantly exacerbates neuroinflammatory responses following stroke, with a senescent-like microglial phenotype and enhanced pathogenicity of innate and adaptive immune cells found in the ischemic brain of aged rodents [47]. It has been emergingly recognized that microglia maintain sex-specific properties in the context of acute focal cerebral ischemia, with female microglia functioning to curtail the evolution of ischemic brain injury [52]. Whether VDR signaling could modulate stroke outcomes in a sex- and age-dependent manner warranted further investigation.

In summary, our study reveals a novel regulatory mechanism by which VDR signaling in microglia/macrophages modulates neuroinflammation and stroke pathogenesis following cerebral ischemia. Specifically, microglia/macrophages lacking VDR not only induces a more primed proinflammatory phenotype in themselves, but also interacts with brain ECs via massive secretion of TNF-α and IFN-γ, thereby facilitating CXCL10 induction and subsequently enhancing peripheral T lymphocytes accumulation. Our findings provide, at least in part, a mechanistic explanation for the detrimental effect of vitamin D deficiency on ischemia brain injury, and highlight functional vitamin D/VDR signaling, which largely relies on a sufficient vitamin D status in clinical practice, as a potential target for the treatment of ischemic stroke.

## Supplementary Information

**Additional file 1: Figure S1.** VDR expression alterations in activated microglia/macrophages after cerebral ischemia. (A, B) Immunofluorescence staining for Iba1 and VDR in the cortex of brain sections from sham mice and in peri-infarct regions on day 1 and 3 post-ischemia. Dashed lines divide the infarction core and ischemic penumbra (*n* = 5 per group). Scale bar, 20 µm. Each symbol represents one mouse. Data are expressed as mean ± SD and analyzed by one-way ANOVA followed by Dunnett's post hoc test. *P < 0.05, **** P < 0.0001. **Figure S2.** VDR elimination in microglia/macrophages exerts little impact on key physiological parameters under normal conditions. (A) Construction (left) and breeding strategies (right) of *Vdr-*cKO and control mice. *Vdr-*cKO mice possess both homozygous loxP-bordered *Vdr* allele and heterozygous *Cx3cr1*^*CreER*^ allele. (B) Immunostaining for Iba1 and VDR in peri-infarct regions of control and *Vdr-*cKO mice 3 days after MCAO, indicating the successful genetic ablation of VDR (*n* = 4 per group). (C) Body weight of control and *Vdr*-cKO mice during 4–12-week development, as measured one week after tamoxifen injection (*n* = 8 per group). (D, E) Quantitative analysis of the counts of splenic immune cells of normal control and *Vdr*-cKO mice by FACS, including CD11b^+^Ly6G^+^ neutrophils, CD11b^+^F4/80^+^ monocytes/macrophages, CD4^+^ and CD8^+^ T lymphocytes, CD3^−^CD19^+^ B lymphocytes, and CD3^−^NK1.1^+^ NK cells (*n* = 3 per group). (F) Representative brain images of normal control and *Vdr*-cKO mice stained for Iba1, GFAP, and NeuN, respectively. The counts of Iba1^+^, GFAP^+^, and NeuN^+^ cells are quantified (*n* = 3 per group). (G) Representative images of rCBF, as monitored by laser Doppler before and during 60-min MCAO. (H) Quantification of rCBF of control and *Vdr*-cKO mice throughout MCAO procedure (*n* = 5 per group). Each symbol represents one mouse. Data are expressed as mean ± SD and analyzed by two-tailed unpaired *t*-test. **** P < 0.0001. **Figure S3.** VDR deficiency in microglia/macrophages exacerbates infarct volumes in female stroke mice. (A) TTC staining of brain sections from female *Vdr-*cKO and control mice 3 days after MCAO. Four representative rostro-caudal brain sections are displayed. Scale bar, 5 mm. (B) Quantitative analysis of infarct volumes of each group in (A) (*n* = 6 per group). Each symbol represents one mouse. Data are expressed as mean ± SD and analyzed by two-tailed unpaired *t*-test. * P < 0.05. **Figure S4.** Gating strategy of immune cell subsets with the CNS. **Figure S5.** VDR deficiency potentiates microglial IFN-γ production in the presence of conditioned media from damaged neurons. (A) Diagram describes the culture conditions for stimulating IFN-γ production of microglia isolated from adult control and *Vdr*-cKO mice 1 day after MCAO. (B) Quantification of IFN-γ concentrations in microglia cultures of indicated groups (*n* = 3 per group). Each symbol represents one biological replicate. Data are expressed as mean ± SD and analyzed by two-tailed unpaired *t*-test. ** P < 0.01. **Figure S6.** The amounts of cerebral and splenic immune cells of control and *Vdr-*cKO mice after cerebral ischemia. (A, B) Representative immunofluorescence images of CD3 in brain sections of control and *Vdr-*cKO mice 3 days after MCAO (*n* = 6 per group). (C, D) Representative images of spleens of indicated groups. Scale bar, 1 cm. Spleen weights and the number of splenocytes of indicated groups are shown in (D) (*n* = 6 per group). Each symbol represents one mouse. Data are expressed as mean ± SD and analyzed by two-tailed unpaired *t*-test. * P < 0.05. **Figure S7.** VDR deletion in microglia/macrophages enhances endothelial CXCL10 expression. (A) Quantification of the fluorescence intensity of CXCL10 in brain sections of control and *Vdr*-cKO mice 3 days after MCAO (*n* = 6 per group). Dashed lines divide the infarction core and ischemic penumbra. Scale bar, 100 µm. (B) Immunostaining for CXCL10 with Iba1, GFAP, NeuN, and CD31 in the ischemic brain of *Vdr*-cKO mice 3 days after MCAO. Scale bar, 20 µm. (C) Immunostaining for CXCL10, CD31, and PDGFRβ of brain sections of indicated groups 3 days after MCAO. Scale bar, 40 µm. Each symbol represents one mouse. Data are expressed as mean ± SD. Two-tailed unpaired Student’s *t*-test was used for (A), **P < 0.01. **Figure S8.** Establishment of differential VDR expression in BV2 microglial cells. (A, B) Western blot analysis of VDR in controls and BV2 cells 72 h after OGD (*n* = 4 per group). (C, D) Western blot analysis of VDR in OGD-treated BV2 cells transfected with lentivirus harboring *Vdr* shRNA (sh*Vdr*) or non-specific control shRNA (shNC) (*n* = 3 per group). (E, F) Western blot analysis of VDR in shNC-BV2 cells incubated with different concentrations of 1,25(OH)_2_D (*n* = 4 per group). Each symbol represents one biological replicate. Data are expressed as mean ± SD. * *P* < 0.05, *** P < 0.001, **** P < 0.0001 by two-tailed unpaired Student’s *t*-test for (B and D) and one-way ANOVA followed by Tukey's post hoc test for (F). **Figure S9.** VDR modulates TNF-α expression and NF-κB signaling in BV2 cell. (A) TNF-α levels in the supernatants of BV2 cells with basic, downregulated and upregulated VDR expression, respectively, 24 h following OGD (*n* = 5 per group). (B, C) Western blot analysis of NF-κB p-p65 subunit in OGD-treated BV2 cells of indicated groups (*n* = 6 per group). (D, E) Immunofluorescence staining for p65 and VDR in OGD-treated BV2 cells of indicated groups. Scale bar, 5 µm. The ratio of nuclear p65 to cytoplasmic p65 is shown in (E). Each symbol represents one biological replicate. Data are expressed as mean ± SD. * *P* < 0.05, **P < 0.01, *** P < 0.001, **** P < 0.0001 by one-way ANOVA followed by Dunnett's post hoc test. **Table S1.** Primers used in qRT-PCR analysis

## Data Availability

All data are available in the main text or the supplementary materials. The original data of this study are accessible from the corresponding author upon rational request. Raw RNA-sequencing data for the 16 samples in this study were uploaded to the Gene Expression Omnibus database (http://www.ncbi.nlm.nih.gov/geo/) under the accession number GSE190171.
