# Supplementary material for: Microglia/macrophages require vitamin D signaling to restrain neuroinflammation and brain injury in a murine ischemic stroke model
Source: J Neuroinflammation. 2023 Mar 8;20:63. doi: 10.1186/s12974-023-02705-0 (PMC9993716; doi:10.1186/s12974-023-02705-0)
Supplement: Supplementary file 1 — Additional file 1: Figure S1. VDR expression alterations in activated microglia/macrophages after cerebral ischemia. (A, B) Immunofluorescence staining for Iba1 and VDR in the cortex of brain sections from sham mice and in peri-infarct regions on day 1 and 3 post-ischemia. Dashed lines divide the infarction core and ischemic penumbra (n = 5 per group). Scale bar, 20 µm. Each symbol represents one mouse. Data are expressed as mean ± SD and analyzed by one-way ANOVA followed by Dunnett's post hoc test. *P < 0.05, **** P < 0.0001. Figure S2. VDR elimination in microglia/macrophages exerts little impact on key physiological parameters under normal conditions. (A) Construction (left) and breeding strategies (right) of Vdr-cKO and control mice. Vdr-cKO mice possess both homozygous loxP-bordered Vdr allele and heterozygous Cx3cr1CreER allele. (B) Immunostaining for Iba1 and VDR in peri-infarct regions of control and Vdr-cKO mice 3 days after MCAO, indicating the successful genetic ablation of VDR (n = 4 per group). (C) Body weight of control and Vdr-cKO mice during 4–12-week development, as measured one week after tamoxifen injection (n = 8 per group). (D, E) Quantitative analysis of the counts of splenic immune cells of normal control and Vdr-cKO mice by FACS, including CD11b+Ly6G+ neutrophils, CD11b+F4/80+ monocytes/macrophages, CD4+ and CD8+ T lymphocytes, CD3−CD19+ B lymphocytes, and CD3−NK1.1+ NK cells (n = 3 per group). (F) Representative brain images of normal control and Vdr-cKO mice stained for Iba1, GFAP, and NeuN, respectively. The counts of Iba1+, GFAP+, and NeuN+ cells are quantified (n = 3 per group). (G) Representative images of rCBF, as monitored by laser Doppler before and during 60-min MCAO. (H) Quantification of rCBF of control and Vdr-cKO mice throughout MCAO procedure (n = 5 per group). Each symbol represents one mouse. Data are expressed as mean ± SD and analyzed by two-tailed unpaired t-test. **** P < 0.0001. Figure S3. VDR deficiency in microglia/ma [file 12974_2023_2705_MOESM1_ESM.docx]

**Microglia/macrophages require vitamin D signaling to restrain neuroinflammation and brain injury in a murine ischemic stroke model**

Pan Cui^1,2,3†^, Wanting Lu^1,3†^, Junjie Wang^4†^, Fei Wang^1,3^, Xiyue Zhang^1,3^, Xiaodan Hou^1,3^, Fang Xu ^1,3^, Yan Liang^1,3^, Guoliang Chai^1,3,5*^, and Junwei Hao^1,3,5*^

**Author affiliations:**

1. Department of Neurology, Xuanwu Hospital, Capital Medical University, National Center for Neurological Disorders, Beijing, 100053, China
2. Department of Neurology, The First Affiliated Hospital of Zhengzhou University, Zhengzhou, Henan, 450007, China
3. Beijing Municipal Geriatric Medical Research Center, Beijing, China
4. Department of Neurology, Tianjin Neurological Institute, Tianjin Medical University General Hospital, Tianjin 300052, China
5. Key Laboratory for Neurodegenerative Diseases of Ministry of Education, Beijing, China

*Corresponding author. E-mail: haojunwei@vip.163.com (J.H.); guoliang.chai@xwh.ccmu.edu.cn (G.C.)

†These authors contributed equally to this work.

# Abstract

Vitamin D deficiency is associated with worse clinical outcomes after ischemic stroke; nevertheless, the pathophysiological mechanisms remain largely unexplored. In this study, we characterized the molecular mechanisms of how vitamin D signaling modulated stroke progression in male mouse ischemia-reperfusion stroke models. We found that vitamin D receptor (VDR) exhibited a predominant upregulation in peri-infarct microglia/macrophages following cerebral ischemia. Conditional *Vdr* inactivation in microglia/macrophages markedly augmented infarct volumes and neurological deficits. VDR-deficient microglia/macrophages exhibited a more primed proinflammatory phenotype with substantial secretion of TNF-α and IFN-γ. These inflammatory cytokines further enhanced CXCL10 release from endothelial cells and blood-brain barrier disruption, and ultimately infiltration of peripheral T lymphocytes. Notably, blocking TNF-α and IFN-γ significantly ameliorated stroke phenotypes in *Vdr* conditional knockout mice. Collectively, VDR signaling in microglia/macrophages plays a crucial role in restraining ischemia-elicited neuroinflammation and stroke progression. Our findings delineate a novel mechanism underlying the association between vitamin D deficiency and poor stroke outcomes, and underline the significance of maintaining a functional vitamin D signaling in the management of acute ischemic stroke.

**Keywords:** acute ischemic stroke; vitamin D deficiency; vitamin D receptor; microglia/macrophages; neuroinflammation


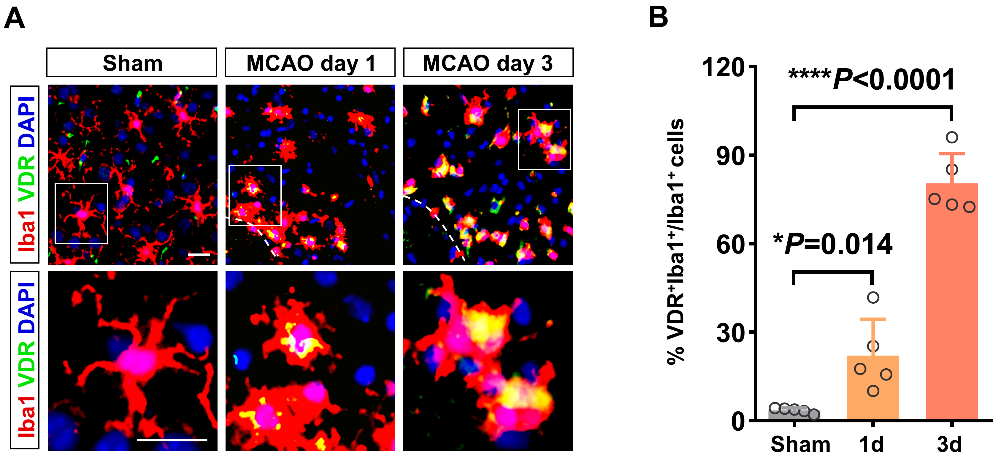


**Figure S1. VDR expression alterations in activated microglia/macrophages after cerebral ischemia. (A, B)** Immunofluorescence staining for Iba1 and VDR in the cortex of brain sections from sham mice and in peri-infarct regions on day 1 and 3 post-ischemia. Dashed lines divide the infarction core and ischemic penumbra (*n* = 5 per group). Scale bar, 20 µm. Each symbol represents one mouse. Data are expressed as mean ± SD and analyzed by one-way ANOVA followed by Dunnett's post hoc test. **P* < 0.05, **** *P* < 0.0001.


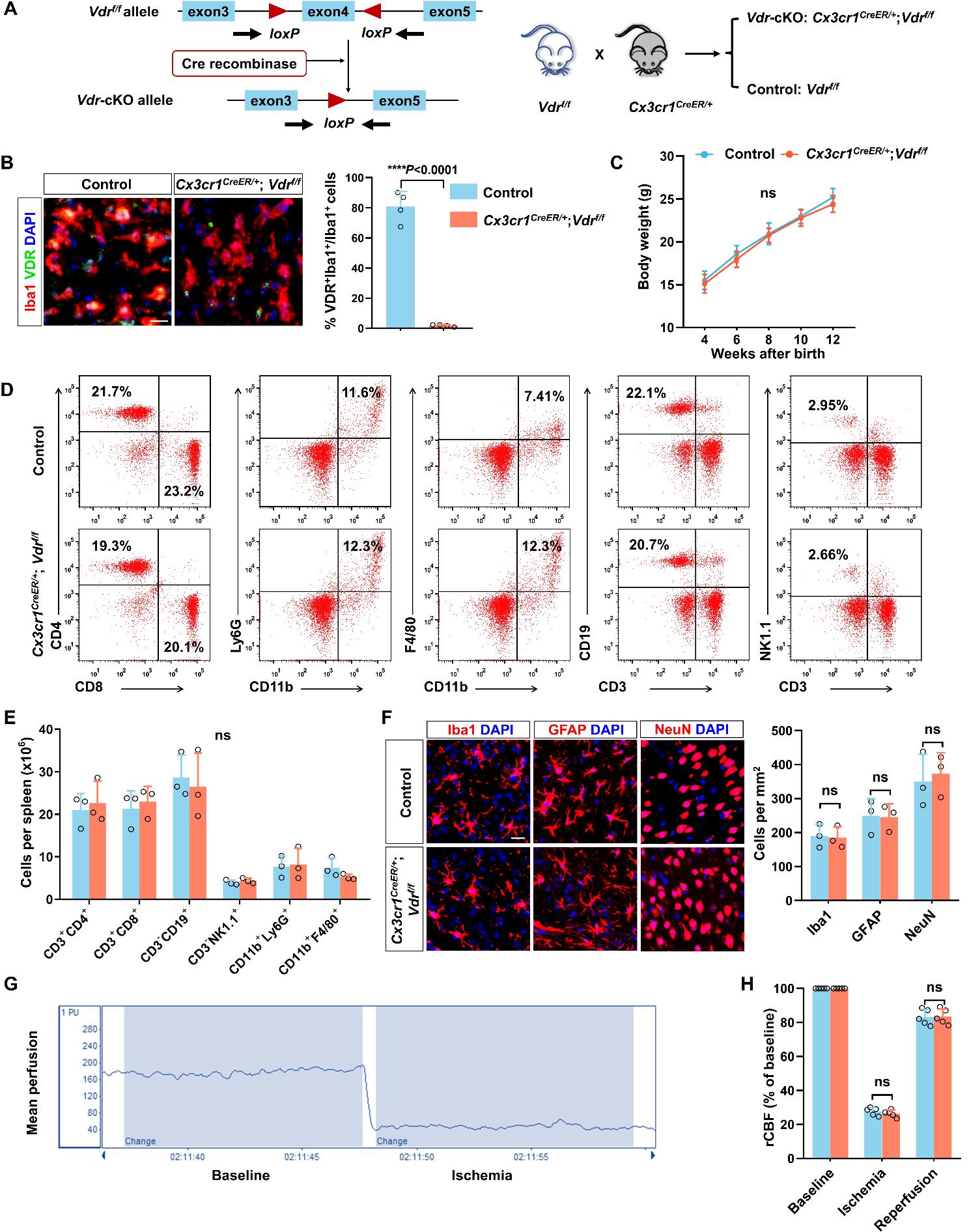


**Figure S2. VDR elimination in microglia/macrophages exerts little impact on key physiological parameters under normal conditions. (A)** Construction (left) and breeding strategies (right) of *Vdr-*cKO and control mice. *Vdr-*cKO mice possess both homozygous loxP-bordered *Vdr* allele and heterozygous *Cx3cr1^CreER^* allele. **(B)** Immunostaining for Iba1 and VDR in peri-infarct regions of control and *Vdr-*cKO mice 3 days after MCAO, indicating the successful genetic ablation of VDR (*n =* 4 per group). **(C)** Body weight of control and *Vdr*-cKO mice during 4-12-week development, as measured one week after tamoxifen injection (*n =* 8 per group). **(D, E)** Quantitative analysis of the counts of splenic immune cells of normal control and *Vdr*-cKO mice by FACS, including CD11b^+^Ly6G^+^ neutrophils, CD11b^+^F4/80^+^ monocytes/macrophages, CD4^+^ and CD8^+^ T lymphocytes, CD3^-^CD19^+^ B lymphocytes, and CD3^-^NK1.1^+^ NK cells (*n =* 3 per group). **(F)** Representative brain images of normal control and *Vdr*-cKO mice stained for Iba1, GFAP, and NeuN, respectively. The counts of Iba1^+^, GFAP^+^, and NeuN^+^ cells are quantified (*n =* 3 per group). **(G)** Representative images of rCBF, as monitored by laser Doppler before and during 60-min MCAO. **(H)** Quantification of rCBF of control and *Vdr*-cKO mice throughout MCAO procedure (*n =* 5 per group). Each symbol represents one mouse. Data are expressed as mean ± SD and analyzed by two-tailed unpaired t-test. **** *P* < 0.0001.


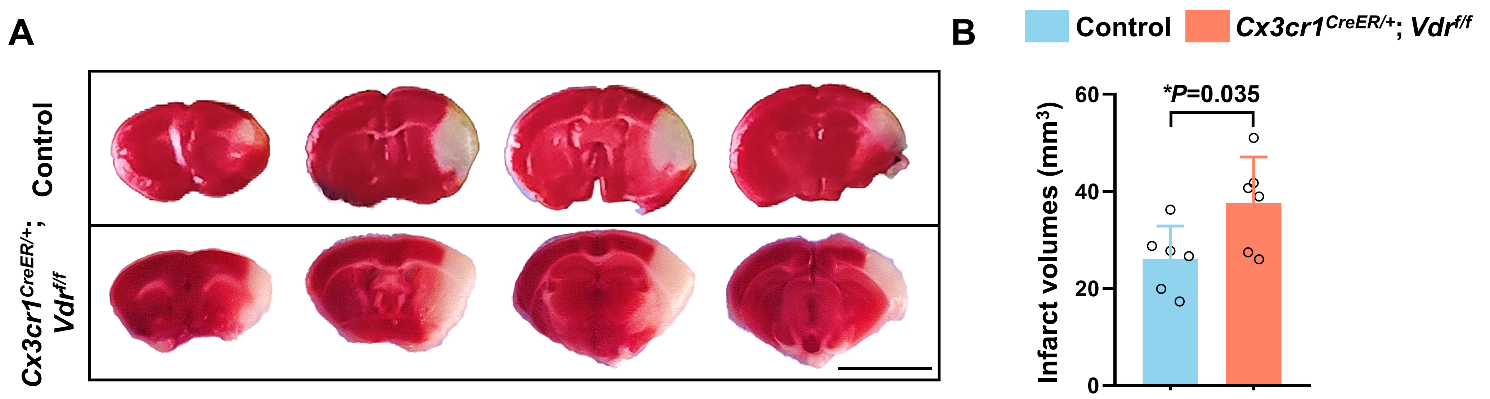


**Figure S3. VDR deficiency in microglia/macrophages exacerbates infarct volumes in female stroke mice. (A)** TTC staining of brain sections from female *Vdr-*cKO and control mice 3 days after MCAO. Four representative rostro-caudal brain sections are displayed. Scale bar, 5 mm. **(B)** Quantitative analysis of infarct volumes of each group in (A) (*n =* 6 per group). Each symbol represents one mouse. Data are expressed as mean ± SD and analyzed by two-tailed unpaired t-test. * *P* < 0.05.


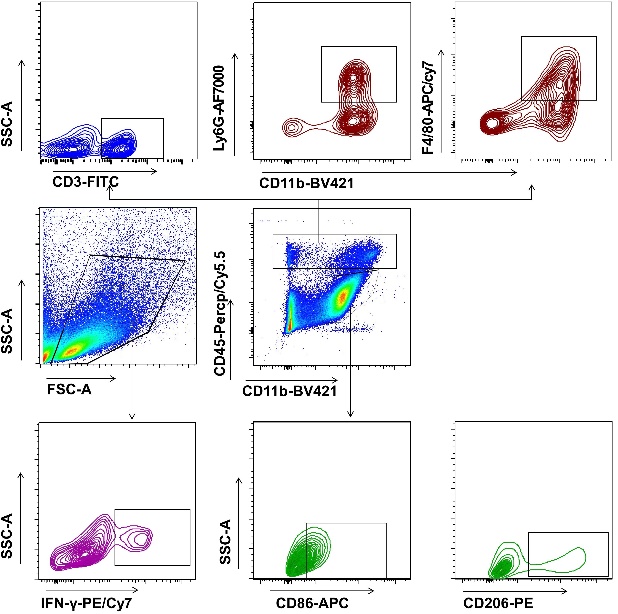


**Figure S4. Gating strategy of immune cell subsets with the CNS.**


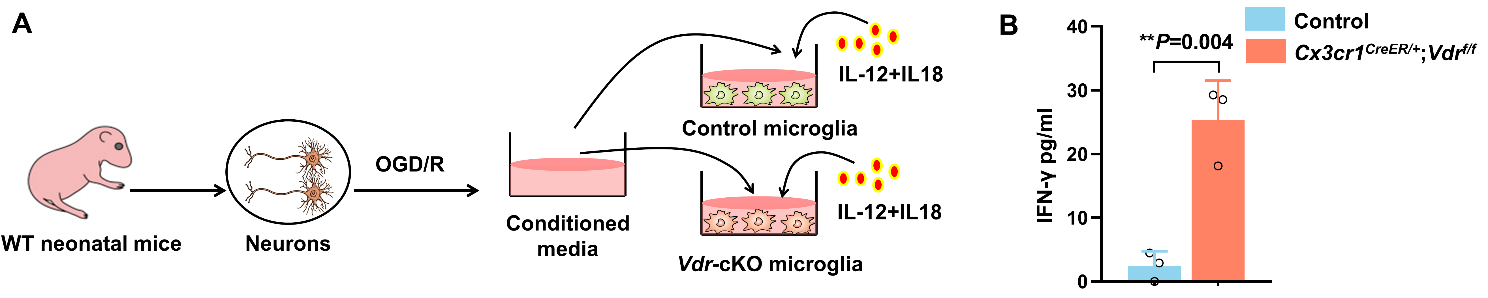


**Figure S5. VDR deficiency potentiates microglial IFN-γ production in the presence of conditioned media from damaged neurons. (A)** Diagram describes the culture conditions for stimulating IFN-γ production of microglia isolated from adult control and *Vdr*-cKO mice 1 day after MCAO. **(B)** Quantification of IFN-γ concentrations in microglia cultures of indicated groups (*n* = 3 per group). Each symbol represents one biological replicate. Data are expressed as mean ± SD and analyzed by two-tailed unpaired t-test. ** *P* < 0.01.


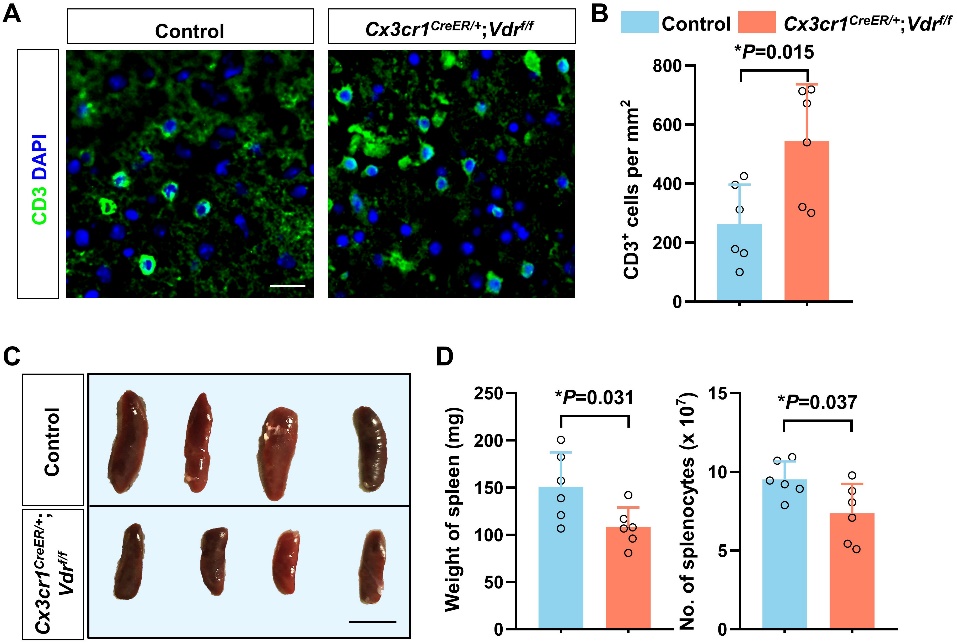


**Figure S6. The amounts of cerebral and splenic immune cells of control and *Vdr-*cKO mice after cerebral ischemia.** **(A, B)** Representative immunofluorescence images of CD3 in brain sections of control and *Vdr-*cKO mice 3 days after MCAO (*n* = 6 per group). **(C, D)** Representative images of spleens of indicated groups. Scale bar, 1 cm. Spleen weights and the number of splenocytes of indicated groups are shown in (D) (*n* = 6 per group). Each symbol represents one mouse. Data are expressed as mean ± SD and analyzed by two-tailed unpaired t-test. * *P* < 0.05.


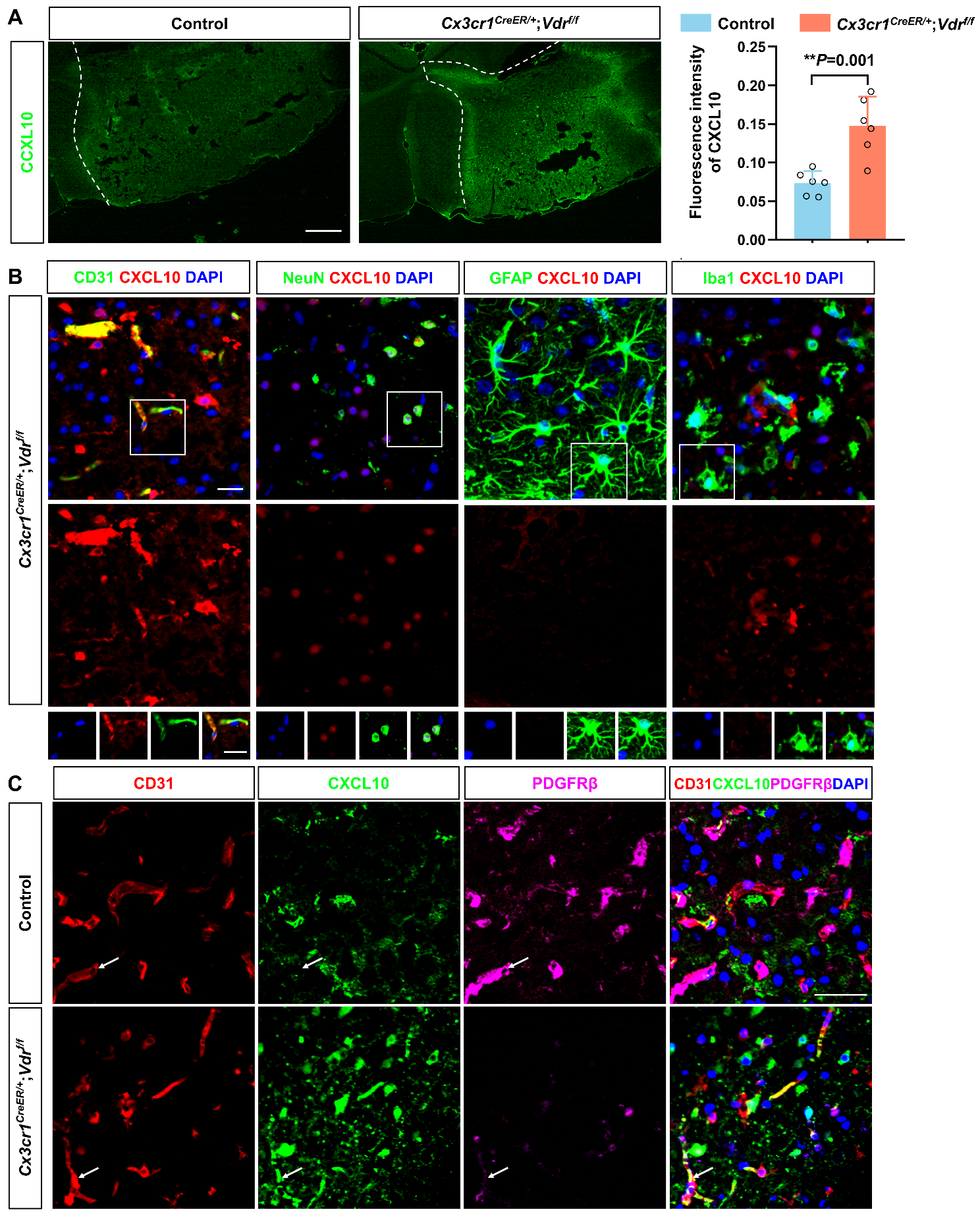


**Figure S7. VDR deletion in microglia/macrophages enhances endothelial CXCL10 expression. (A)** Quantification of the fluorescence intensity of CXCL10 in brain sections of control and *Vdr*-cKO mice 3 days after MCAO (*n* = 6 per group). Dashed lines divide the infarction core and ischemic penumbra. Scale bar, 100 µm. **(B)** Immunostaining for CXCL10 with Iba1, GFAP, NeuN, and CD31 in the ischemic brain of *Vdr*-cKO mice 3 days after MCAO. Scale bar, 20 µm. **(C)** Immunostaining for CXCL10, CD31, and PDGFRβ of brain sections of indicated groups 3 days after MCAO. Scale bar, 40 µm. Each symbol represents one mouse. Data are expressed as mean ± SD. Two-tailed unpaired Student’s t-test was used for (A), ***P* < 0.01.


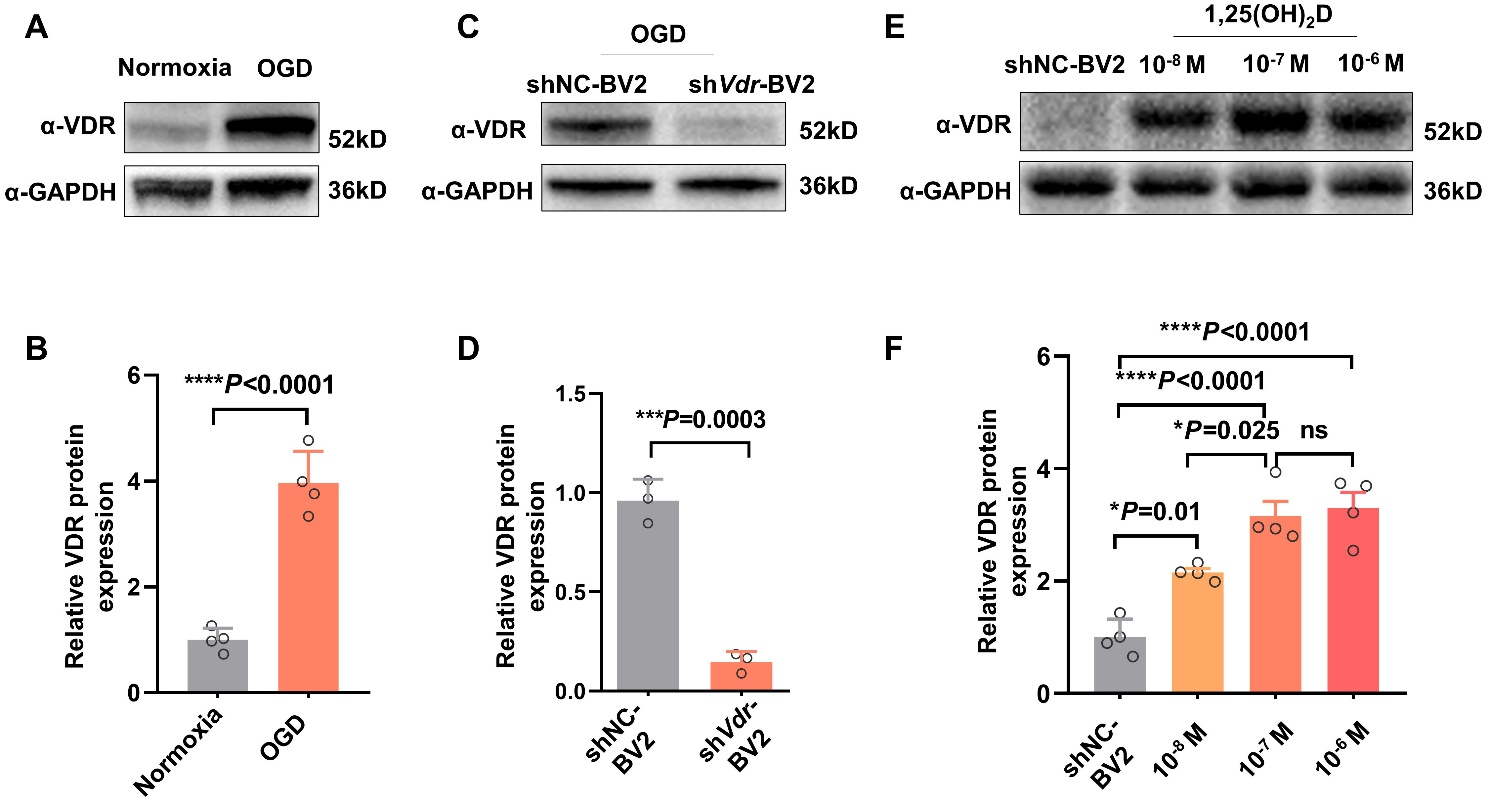


**Figure S8. Establishment of differential VDR expression in BV2 microglial cells. (A, B)** Western blot analysis of VDR in controls and BV2 cells 72 h after OGD (*n* = 4 per group). **(C, D)** Western blot analysis of VDR in OGD-treated BV2 cells transfected with lentivirus harboring *Vdr* shRNA (sh*Vdr*) or non-specific control shRNA (shNC) (*n* = 3 per group). **(E, F)** Western blot analysis of VDR in shNC-BV2 cells incubated with different concentrations of 1,25(OH)_2_D (*n* = 4 per group). Each symbol represents one biological replicate. Data are expressed as mean ± SD. * *P* < 0.05, *** *P* < 0.001, **** *P* < 0.0001 by two-tailed unpaired Student’s t-test for (B and D) and one-way ANOVA followed by Tukey's post hoc test for (F).


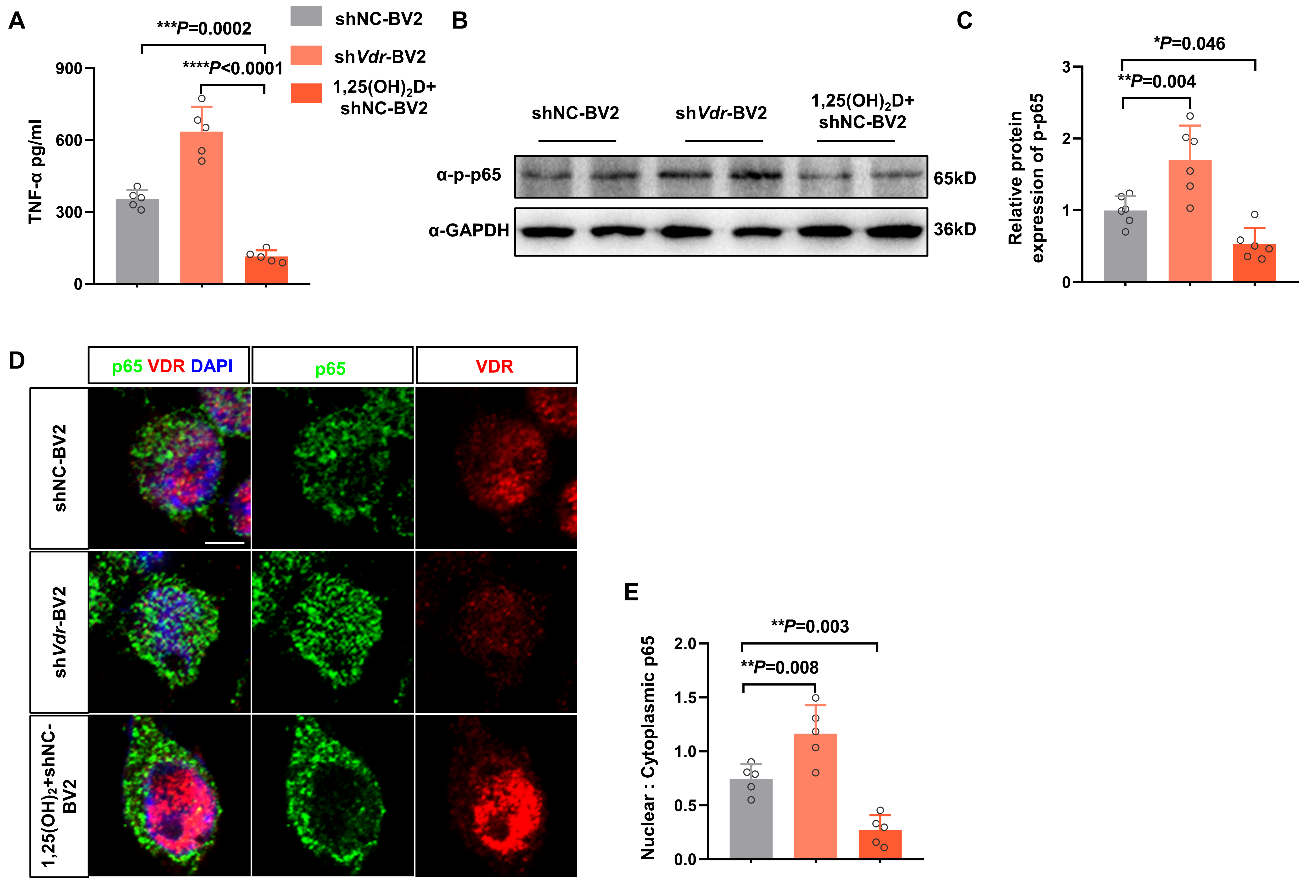


**Figure S9. VDR modulates TNF-α expression and NF-κB signaling in BV2 cell. (A)** TNF-α levels in the supernatants of BV2 cells with basic, downregulated and upregulated VDR expression, respectively, 24 h following OGD (*n =* 5 per group). **(B, C)** Western blot analysis of NF-κB p-p65 subunit in OGD-treated BV2 cells of indicated groups (*n =* 6 per group). **(D, E)** Immunofluorescence staining for p65 and VDR in OGD-treated BV2 cells of indicated groups. Scale bar, 5 µm. The ratio of nuclear p65 to cytoplasmic p65 is shown in (E). Each symbol represents one biological replicate. Data are expressed as mean ± SD. * *P* < 0.05, ***P* < 0.01, *** *P* < 0.001, **** *P* < 0.0001 by one-way ANOVA followed by Dunnett's post hoc test.

# Table S1. Primers used in qRT-PCR analysis

| Gene | Forward | Reverse |
| --- | --- | --- |
| *Gapdh* | GCCAAGGCTGTGGGCAAGGT | TCTCCAGGCGGCACGCAGA |
| *Vdr* | CCTGGTGACTTTGACCGGA | GCCTTCACAGGTCATAGCGT |
| *Tnf-α* | CTGTGAAGGGAATGGGTGTT | GGTCACTGTCCCAGCATCTT |
| *Il-6* | TCCATCCAGTTGCCTTCTTG | GGTCTGTTGGGAGTGGTATC |
| *Il-1β* | GAGTGTGGATCCCAAGCAAT | TACCAGTTGGGGAACTCTGC |
| *Ifn-γ* | GCAACAGCAAGGCGAAAAAG | CGCTTCCTGAGGCTGGATTC |
| *Il-4* | ACCTTGCTGTCACCCTGTTC | GTGAGTTCAGACCGCTGACA |
| *Il-10* | CCAAGCCTTATCGGAAATGA | GGGTCCGACAGCACGAGGCT |
| *Ccl2* | ACGCTTCTGGGCCTGTTGTT | CCTGCTGCTGGTGATTCTCT |
| *Cxcl10* | AAGCTATGTGGAGGTGCGAC | AACCCCTTGGGAAGATGGTG |
| *Cxcl9* | GTGGAGTTCGAGGAACCCTAG | ATTGGGGCTTGGGGCAAAC |
| *Ccl5* | GTGCTCCAATCTTGCAGTAG | GGATTACTGAGTGGCATCCCC |
| *Cd16* | TTTGGACACCCAGATGTTTCAG | GTCTTCCTTGAGCACCTGGATC |
| *Cd32* | AATCCTGCCGTTCCTACTGATC | GTGTCACCGTGTCTTCCTTGAG |
| *Cd206* | CAAGGAAGGTTGGCATTTGT | CCTTTCAGTCCTTTGCAAGC |
| *Cxcr3* | AATGCCACCCATTGCCAGTAC | AGCAGTAGGCCATGACCAGAAG |
| *Vcam1* | GGTATCCCATCACTTGAGCAGG | TGAACCCAAACAGAGGCAGAGT |
| *Il-12* | AAATGAAGCTCTGCATCCTGC | TCACCCTGTTGATGGTCACG |
| *Il-18* | ACTTTGGCCGACTTCACTGT | GGGTTCACTGGCACTTTGAT |
